# Targeting the Mitochondrial Chaperone TRAP1 Alleviates Vascular Pathologies in Ischemic Retinopathy

**DOI:** 10.1002/advs.202302776

**Published:** 2023-11-20

**Authors:** So‐Yeon Kim, Nam Gu Yoon, Jin Young Im, Ji Hye Lee, Juhee Kim, Yujin Jeon, Young Jae Choi, Jong‐Hwa Lee, Akiyoshi Uemura, Dong Ho Park, Byoung Heon Kang

**Affiliations:** ^1^ Department of Biological Sciences Ulsan National Institutes of Science and Technology (UNIST) Ulsan 44919 Republic of Korea; ^2^ SmartinBio Inc. Cheongju 28160 Republic of Korea; ^3^ Department of Ophthalmology, School of Medicine Kyungpook National University, Kyungpook National University Hospital Daegu 41944 Republic of Korea; ^4^ Cell & Matrix Research Institute Kyungpook National University Daegu 41944 Republic of Korea; ^5^ Bioanalysis and Pharmacokinetics Research Group Korea Institute of Toxicology Daejeon 34114 Republic of Korea; ^6^ Department of Human and Environment Toxicology University of Science & Technology Daejeon 34113 Republic of Korea; ^7^ Department of Ophthalmology and Visual Science Nagoya City University Graduate School of Medical Sciences Nagoya 467‐8601 Japan

**Keywords:** calcium, calpain‐1, hypoxia‐inducible factor 1α (HIF1α), ischemic retinopathy, mitochondrial permeability transition pore (mPTP), tumor necrosis factor receptor‐associated protein 1 (TRAP1)

## Abstract

Activation of hypoxia‐inducible factor 1α (HIF1α) contributes to blood‐retinal barrier (BRB) breakdown and pathological neovascularization responsible for vision loss in ischemic retinal diseases. During disease progression, mitochondrial biology is altered to adapt to the ischemic environment created by initial vascular dysfunction, but the mitochondrial adaptive mechanisms, which ultimately contribute to the pathogenesis of ischemic retinopathy, remain incompletely understood. In the present study, it is identified that expression of mitochondrial chaperone tumor necrosis factor receptor‐associated protein 1 (TRAP1) is essential for BRB breakdown and pathologic retinal neovascularization in mouse models mimicking ischemic retinopathies. Genetic *Trap1* ablation or treatment with small molecule TRAP1 inhibitors, such as mitoquinone (MitoQ) and SB‐U015, alleviate retinal pathologies via proteolytic HIF1α degradation, which is mediated by opening of the mitochondrial permeability transition pore and activation of calcium‐dependent protease calpain‐1. These findings suggest that TRAP1 can be a promising target for the development of new treatments against ischemic retinopathy, such as retinopathy of prematurity and proliferative diabetic retinopathy.

## Graphical abstract

This study reveals that the mitochondrial chaperone TRAP1 is vital for stabilizing HIF1α, a key transcription factor in pathologic microvascular changes in ischemic retinopathies. Therefore, inhibiting TRAP1 genetically or pharmacologically alleviates retinal vascular pathologies by inducing HIF1α proteolytic degradation, indicating that TRAP1 can be a promising target for the development of therapies for ischemic retinopathy.

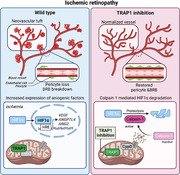

## 1 Introduction

Mitochondria are the metabolic and signaling organelles that sense cellular and environmental changes to reprogram intraorganellar pathways for proper adaptation to pathologies such as ischemia and disrupted energy metabolism.^[^ ^1^ ^]^ Dysregulation of mitochondrial function is a common feature of many human diseases, including cancer and metabolic disorders.^[^ ^1^, ^2^ ^]^ Tumor necrosis factor receptor‐associated protein 1 (TRAP1), a mitochondrial paralog of 90 kDa heat shock protein (Hsp90), reprograms cancer cell metabolism to adapt to the harsh tumor environment.^[^ ^3^ ^]^ In this process, TRAP1 reprograms cellular energetics, cellular redox pathways, calcium homeostasis, and cell death signaling by interacting with the mitochondrial substrate proteins that modulate these processes in cancer cells.^[^ ^3^, ^4^ ^]^ Considering its broad impacts on mitochondrial pathways, TRAP1 could play a role in the onset and progression of human diseases that involve altered mitochondrial function.

The retina is the most metabolically demanding tissue of the body due to its high neuronal density, and thus is vulnerable to energetic insufficiencies caused by ischemia, changes in the availability of metabolic substrates, and mitochondrial dysfunction.^[^ ^5^ ^]^ Adaptive responses involving mitochondrial pathways are necessary to maintain the function of retinal cells, especially under stress conditions.^[^ ^5^, ^6^ ^]^ Retinal ischemia and metabolic stress contribute to the pathologies of devastating sight‐threatening diseases such as retinopathy of prematurity (ROP) and proliferative diabetic retinopathy (PDR).^[^ ^7^ ^]^ Thus, we speculated that TRAP1 could be dysregulated in these disease conditions, reprogramming mitochondrial pathways as a maladaptive compensatory response in ischemic retinopathies, although the role of TRAP1 in these contexts has not been reported.

Cellular adaptation to low oxygen levels involves the stabilization of hypoxia‐inducible factors including HIF1α, which is regulated by the oxygen‐sensing enzyme prolyl hydroxylase (PHD).^[^ ^8^ ^]^ However, retinal ischemia, a common causative mechanism of ocular disease, prompts compensatory stabilization of HIF1α and subsequent elevation of angiogenic and vascular permeability factors such as vascular endothelial growth factor (VEGF), which contribute to retinal vascular hyperpermeability and pathological neovascularization.^[^ ^9^ ^]^ Therefore, intravitreal injection of anti‐VEGF therapies is effective in treating neovascularization and pathologic vascular leakage in ROP and PDR.^[^ ^9^, ^10^ ^]^ However, these pathologies can recur after anti‐VEGF therapy in some patients, and anti‐VEGF therapies for the treatment of ROP have raised concerns that anti‐VEGF drugs may cross the blood‐retinal barrier (BRB), potentially posing risks to premature infants.^[^ ^10^, ^11^ ^]^ Furthermore, in DR patients, repeated intravitreal injections are not only painful, but can also cause major vision‐threatening complications such as endophthalmitis.^[^ ^12^ ^]^ These limitations underscore the urgent clinical need to develop alternative therapeutic targets by identifying novel regulatory mechanisms of disease.

In the present study, we identified a novel and previously unreported function of TRAP1 in maintaining HIF1α stability in ischemic retinopathies. Pharmacologic and genetic inhibition of TRAP1 elevated cytoplasmic calcium to activate the calcium‐dependent protease calpain‐1, which triggers HIF1α degradation independently of PHD to suppress BRB breakdown and pathologic retinal neovascularization. Collectively, our findings suggest that TRAP1 could be a promising therapeutic target for the treatment of HIF1α‐dependent vascular diseases, laying the groundwork for future translational studies.

## 2 Results

### 2.1 TRAP1 is Required for Development of Pathologic Microvascular Changes in Retinopathy

**Figure 1:**
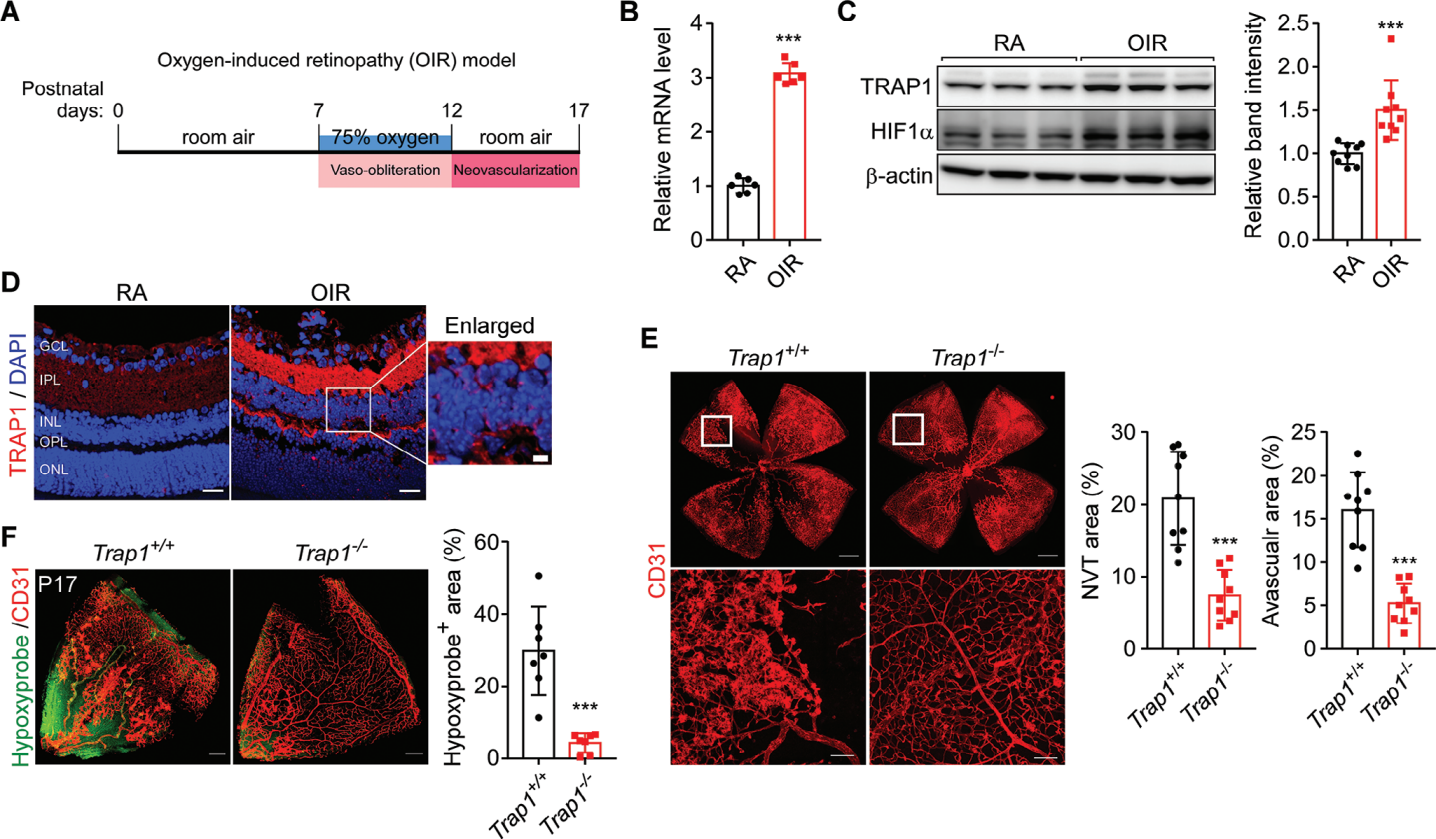
Contribution of TRAP1 to the development of ischemic retinopathy. A) Schematic for the experimental procedures of OIR mice. OIR mice were generated as described in the Experimental Section. P7 mice were exposed to hyperoxia (75% oxygen) for 5 days (vaso‐obliteration), and then returned to room air for 5 days (compensatory neovascularization). At P17, mice were sacrificed for analyses. B) Quantification of TRAP1 mRNA. Retinas collected from OIR mice and age‐matched control mice (RA, room air) were analyzed by quantitative real time PCR (qPCR) (*n* = 6; duplicate experiment of 3 mice/group). C) Quantification of TRAP1 protein levels. Left. RA and OIR mouse retinas were comparatively analyzed by western blotting. Right. The band intensities of TRAP1 were normalized to those of β‐actin in mouse retinas and compared (*n* = 10 mice/group). D) Immunohistochemical staining of TRAP1 in mouse retinas. Mouse retinal sections were stained with an anti‐TRAP1 antibody (red) and DAPI (blue), and analyzed by confocal microscopy. Scale bar, 20 µm and 5 µm (enlarged image). GCL, ganglion cell layer; IPL, inner plexiform layer; INL, inner nuclear layer; OPL, outer plexiform layer; ONL, outer nuclear layer (*n* = 3 mice/RA, *n* = 6 mice/OIR). E) Normalized retinal blood vessels in *Trap1* ^−/−^ OIR mice. Left. Retinas collected from *Trap1* ^+/+^ and *Trap1* ^−/−^ OIR mice were flat‐mounted and stained with an anti‐CD31 antibody. Scale bars, 500 µm (top) and 100 µm (bottom). Right. Quantification of neovascular tuft (NVT) and avascular areas. Retinal vessel images (*n* = 9 mice/group) were quantitatively analyzed as reported previously.^[^ ^13a^ ^]^ F) Hypoxyprobe staining of OIR mouse retinas. Left. Whole‐mounted retinas from OIR mice (P17) were stained with Hypoxyprobe (green) and an anti‐CD31 antibody (red) to visualize hypoxic regions and blood vessels, respectively. Hypoxic areas were significantly decreased in *Trap1* ^−/−^ OIR mice relative to *Trap1* ^+/+^ OIR mice. Scale bar, 200 µm. Right. Quantification of Hypoxyprobe‐positive areas (n = 7 mice/group). Data information: Data are expressed as mean ± SEM. Student *t*‐test, ^***^ *P* < 0.001.

To investigate the involvement of TRAP1 in the pathologic process of ischemic retinopathy, we used the oxygen‐induced retinopathy (OIR) mouse model of ROP^[^ ^13^ ^]^ (**Figure** **1**A). In OIR mice at postnatal day 17 (P17), mRNA and protein levels of TRAP1 were significantly higher than those of age‐matched controls (Figure 1B,C). Immunohistochemical staining of OIR mouse retinas revealed elevated TRAP1 levels throughout the entire areas of the inner plexiform layer (IPL) and outer plexiform layer (OPL) (Figure 1D), which were enriched in mitochondria, as demonstrated by staining for mitochondrial inner membrane proteins (Figure S1, Supporting Information), suggesting mitochondrial alterations in diseased retinal cells.

To investigate the contribution of TRAP1 to retinal vascular pathologies, gene‐trap *Trap1* knockout (KO) mice were generated (Figure S2A,B, Supporting Information) and analyzed in the OIR model. In *Trap1* ^−/−^ OIR retinas, both neovascular tuft formation and vaso‐obliteration were considerably lower than in *Trap1* ^+/+^ OIR mice (Figure 1E), indicating *Trap1* ablation decreased retinal vascular pathogenesis. Consequently, the Hypoxyprobe‐positive area was significantly smaller in *Trap1* ^−/−^ OIR mice than in *Trap1* ^+/+^ OIR mice (Figure 1F). Meanwhile, the retinal vascular structures of *Trap1* ^+/+^ and *Trap1* ^−/−^ mice were comparable at P5, P12, and P36 (Figure S3A–C, Supporting Information) and at P12 in OIR mice (Figure S3D, Supporting Information), demonstrating that TRAP1 deletion alone did not affect normal physiological vascular development or hyperoxic vessel regression in neonatal mouse retinas.

### 2.2 TRAP1‐HIF1α Crosstalk is Essential for the Pathogenesis of Ischemic Retinopathy

**Figure 2:**
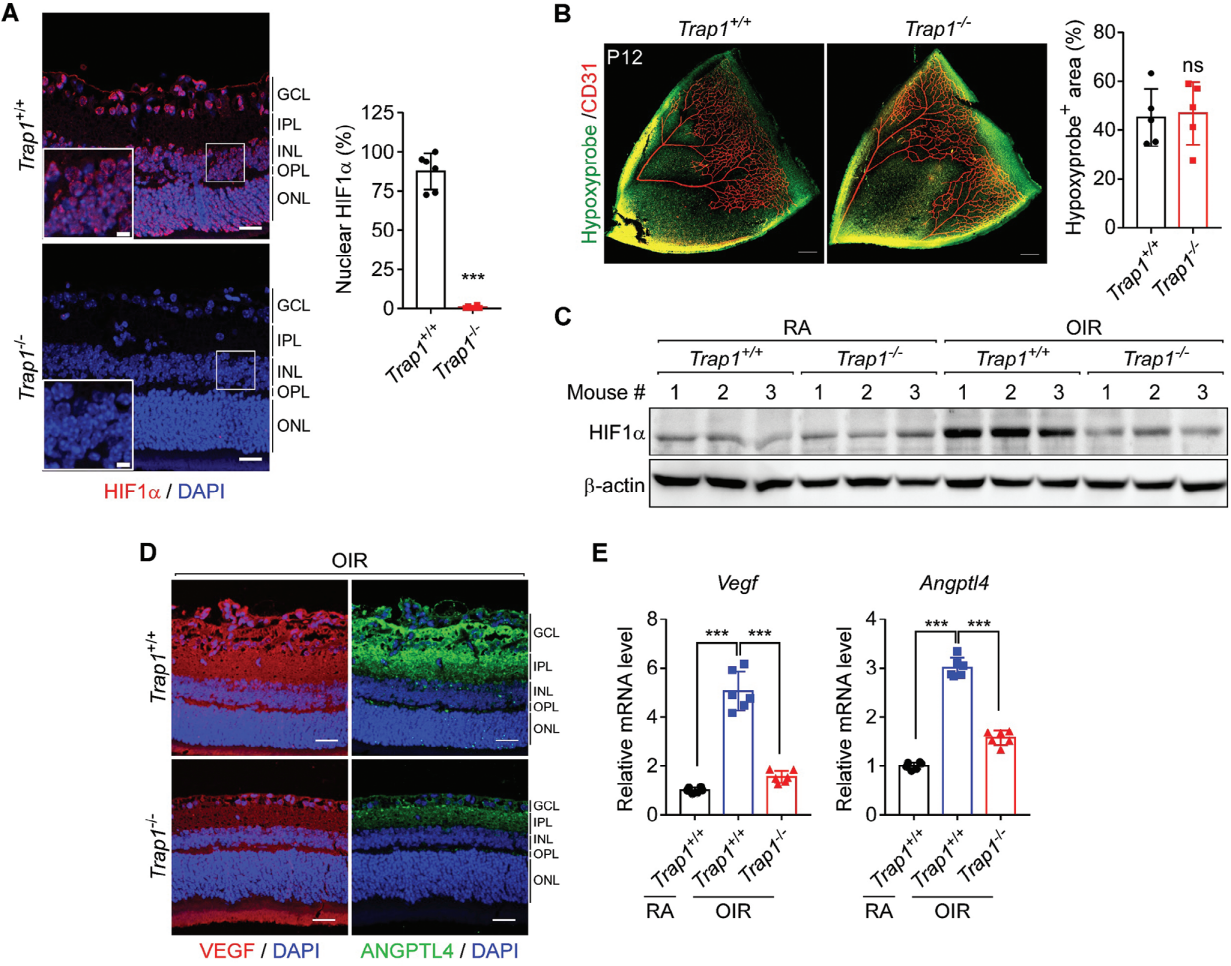
Regulation of HIF1α stability and angiogenic factor expression by TRAP1. A) HIF1α expression in P17 OIR mouse retinas. Left. Retinal sections collected from *Trap1* ^+/+^ and *Trap1* ^−/−^ OIR mice were stained with an anti‐HIF1α antibody (red) and DAPI (blue), and imaged by confocal microscopy. Scale bars, 20 µm and 5 µm (inset). Right Quantification of the ratio (%) of HIF1α/DAPI double‐positive areas to DAPI‐positive areas (*n* = 6 mice/group). B) Identification of hypoxic areas in P12 OIR mouse retinas. Left. Whole‐mount retinas were stained with Hypoxyprobe (green) and an anti‐CD31 antibody (red) to visualize hypoxic regions and blood vessels, respectively. Scale bar, 200 µm. Right. Quantification of hypoxic areas. Hypoxyprobe‐positive areas were quantified from the obtained images (*n* = 5 mice/group). C) Expression of HIF1α in P12 control (RA) and OIR mouse retinas. Western blot analysis was performed to measure HIF1α expression in the retinas of *Trap1* ^+/+^ and *Trap1* ^−/−^ RA and OIR mice (*n* = 3 mice/group). D) VEGF and ANGPTL4 expression in mouse P17 OIR retinas. Retinal sections from *Trap1* ^+/+^ and *Trap1* ^−/−^ OIR mice were stained with anti‐VEGF (red) and anti‐ANGPTL4 (green) antibodies, and analyzed by confocal microscopy (*n* = 3 mice/group). Scale bar, 20 µm. E) Quantitation of *Vegf* and *Angptl4* mRNAs in P17 OIR mouse retinas. mRNA levels in control (RA) and OIR *Trap1* ^+/+^ and *Trap1* ^−/−^ mouse retinas were analyzed by qPCR and compared (*n* = 6; duplicate experiment of 3 mice/group). Data information: Data are expressed as mean ± SEM. Student *t*‐test, ^***^ *P* < 0.001; ns, not significant.

OIR mice exhibited retinal hypoxia (Figure 1F) and elevated expression of HIF1α (Figure 1C), a master transcription factor that upregulates the expression of key angiogenic factors such as VEGF and angiopoietin‐like 4 (ANGPTL4) to induce aberrant retinal angiogenesis.^[^ ^15^ ^]^ Retinal expression and nuclear localization of HIF1α were much lower in *Trap1* ^−/−^ OIR mice than in *Trap1* ^+/+^ OIR mice (**Figure** **2**A). At 6 h after transfer from a hyperoxic chamber to room air, the level of Hypoxyprobe staining was comparably strong in OIR *Trap*1^+/+^ and *Trap*1^−/−^ mice at P12 (Figure 2B), indicating that retinal hypoxia occurs in OIR mice at P12 regardless of their TRAP1 status. However, only the *Trap1* ^−/−^ retinas exhibited HIF1α degradation (Figure 2C), suggesting that TRAP1 ablation promoted oxygen‐independent degradation of HIF1α. Consequently, expression of the angiogenic factors VEGF and ANGPTL4 was reduced in *Trap1* ^−/−^ OIR mice (Figure 2D,E). Interestingly, knockout of TRAP1expression in OIR mice decreased angiogenic factor levels to those similar to control mice, but without completely eliminating their expression (Figure 2E).

The strong positive VEGF staining of glutamine synthetase‐positive cells (Figure S4A, Supporting Information) suggested that Müller cells express angiogenic factors, as previously reported,^[^ ^14^, ^16^ ^]^ In primary mouse Müller cells and the human Müller cell line MIO‐M1 exposed to hypoxia, siRNA‐mediated depletion of TRAP1 induced degradation of HIF1α protein without affecting its mRNA level and reduced expression of HIF1α‐regulated angiogenic factors (Figure S4B–E, Supporting Information). Further, HIF1α expression also increased TRAP1 expression in Müller cells subjected to hypoxia (Figure S5A–C, Supporting Information), as reported previously,^[^ ^17^ ^]^ indicating positive reciprocal regulation exists between HIF1α and TRAP1. Collectively, these data indicate that TRAP1 is essential for HIF1α stabilization and subsequent angiogenic factor expression in the hypoxic retina.

### 2.3 TRAP1‐Dependent Stabilization of HIF1α in a Diabetic Mouse Model

**Figure 3:**
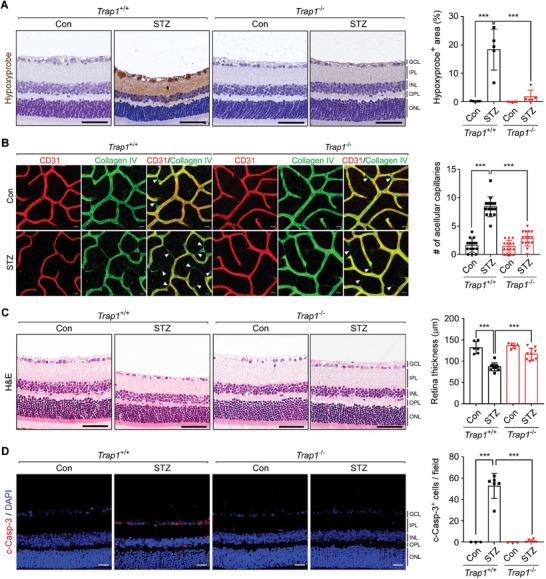
*Trap1* KO decreases vascular abnormalities in STZ mice. A) Hypoxyprobe staining of the retinas of 16‐week post‐STZ injection *Trap1* ^+/+^ and *Trap1* ^−/−^ mice of 25 weeks old (Figure S6A, Supporting information). Left. Retinal sections of *Trap1* ^+/+^ and *Trap1* ^−/−^ STZ mice were analyzed by Hypoxyprobe staining. Scale bar, 50 µm. Right. Quantitation of Hypoxyprobe‐positive areas (*n* = 5 mice/group). B) Decreased retinal capillary degeneration in *Trap1* ^−/−^ STZ mice. Left. STZ and control (Con) retinas from *Trap1* ^+/+^ and *Trap1* ^−/−^ mice were flat‐mounted and stained with anti‐CD31 (red) and anti‐collagen IV antibodies (green) to visualize blood vessels and basement membranes, respectively. White triangles indicate acellular capillaries. Scale bar, 20 µm. Right. Quantification of acellular capillaries. The number of acellular capillaries was counted in each microscopic field (*n* = 15; 5 mice/group, 3 fields/mouse). C) Thinning of STZ mouse retinas. Left. Retinal sections collected from *Trap1* ^+/+^ and *Trap1* ^−/−^ STZ mice were analyzed by H&E staining. Scale bar, 50 µm. Right. Quantification of retinal thickness (control n = 6, STZ *n* = 10 mice/group). D) Cell death in STZ mouse retinas. Left. Retinal sections of *Trap1* ^+/+^ and *Trap1* ^−/−^ STZ mice were analyzed by cleaved caspase‐3 (c‐Casp‐3, red) staining. Scale bar, 20 µm. Right. Quantification of c‐Casp‐3‐positive cells in STZ mouse retinas (control *n* = 3, STZ *n* = 6 mice/group). Data information: Data are expressed as mean ± SEM. Student *t*‐test, ^***^ *P* < 0.001.

Retinal hypoxia, activation of HIF1α, and consequent expression of angiogenic factors are crucial for the development of diabetic retinopathy.^[^ ^15^, ^18^ ^]^ Thus, to further investigate TRAP1‐dependent retinal vascular changes in vivo, retinal hypoxia and HIF1α stabilization were examined in a streptozotocin (STZ)‐induced mouse model of type 1 diabetes^[^ ^19^ ^]^ (Figure S6A,B, Supporting Information). Despite the lack of noticeable neovascularization, STZ mice exhibited severe retinal hypoxia, as revealed by Hypoxyprobe staining (**Figure** **3**A), and increased TRAP1 and HIF1α expression (Figure S6C–E, Supporting Information), indicating hypoxia in the retinas of these mice similar to that in the retinas of OIR mice. Consistent with the observations in *Trap1* ^−/−^ OIR mice, the nuclear expression of retinal HIF1α and subsequent expression of VEGF and ANGPTL4 were significantly reduced to physiological levels in *Trap1* ^−/−^ STZ mice (Figure S7A–C, Supporting Information). Interestingly, the retinas of diabetic *Trap1* ^−/−^ STZ mice showed significantly lower capillary degeneration and subsequently higher vascular densities than those of *Trap1* ^+/+^ STZ mice (Figure 3B; Figure S7D, Supporting Information).

Furthermore, it has been reported that retinal abnormalities, such as glial dysfunction, immune cell activation, vascular degeneration, and increased cell death, begin to develop even before massive vascular abnormalities appear in both DR rodent models and human patients.^[^ ^10^, ^14^ ^]^ In *Trap1* ^+/+^ STZ mice, Müller glial cells were activated, as evidenced by elevated glial fibrillary acidic protein (GFAP) expression, whereas in *Trap1^−^* ^/−^ STZ mice their activation was suppressed (Figure S8A, Supporting Information). Immune activation, characterized by an increase in the population of activated amoeboid microglia with elevated pro‐inflammatory cytokine expression, was observed in *Trap1* ^+/+^ STZ mice; this activation was completely reversed in *Trap1* ^−/−^ STZ mice (Figure S8B,C, Supporting Information). Furthermore, retinal thickness, indicative of retinal neurodegeneration in diabetes,^[^ ^20^ ^]^ was decreased in *Trap1* ^+/+^, but not in *Trap1* ^−/−^, STZ mice (Figure 3C). Consistently, the massive retinal cell death and increased hypoxia observed in *Trap1* ^+/+^ STZ mice were dramatically reversed in *Trap1* ^−/−^ STZ mice (Figure 3A,D). Collectively, the data strongly suggest that TRAP1 contributes to the development of retinal abnormalities in type 1 diabetic STZ mice, and that loss of TRAP1 reverses these abnormalities in vivo.

### 2.4 TRAP1 Inhibition Restores the BRB in Ischemic Retinopathy

**Figure 4:**
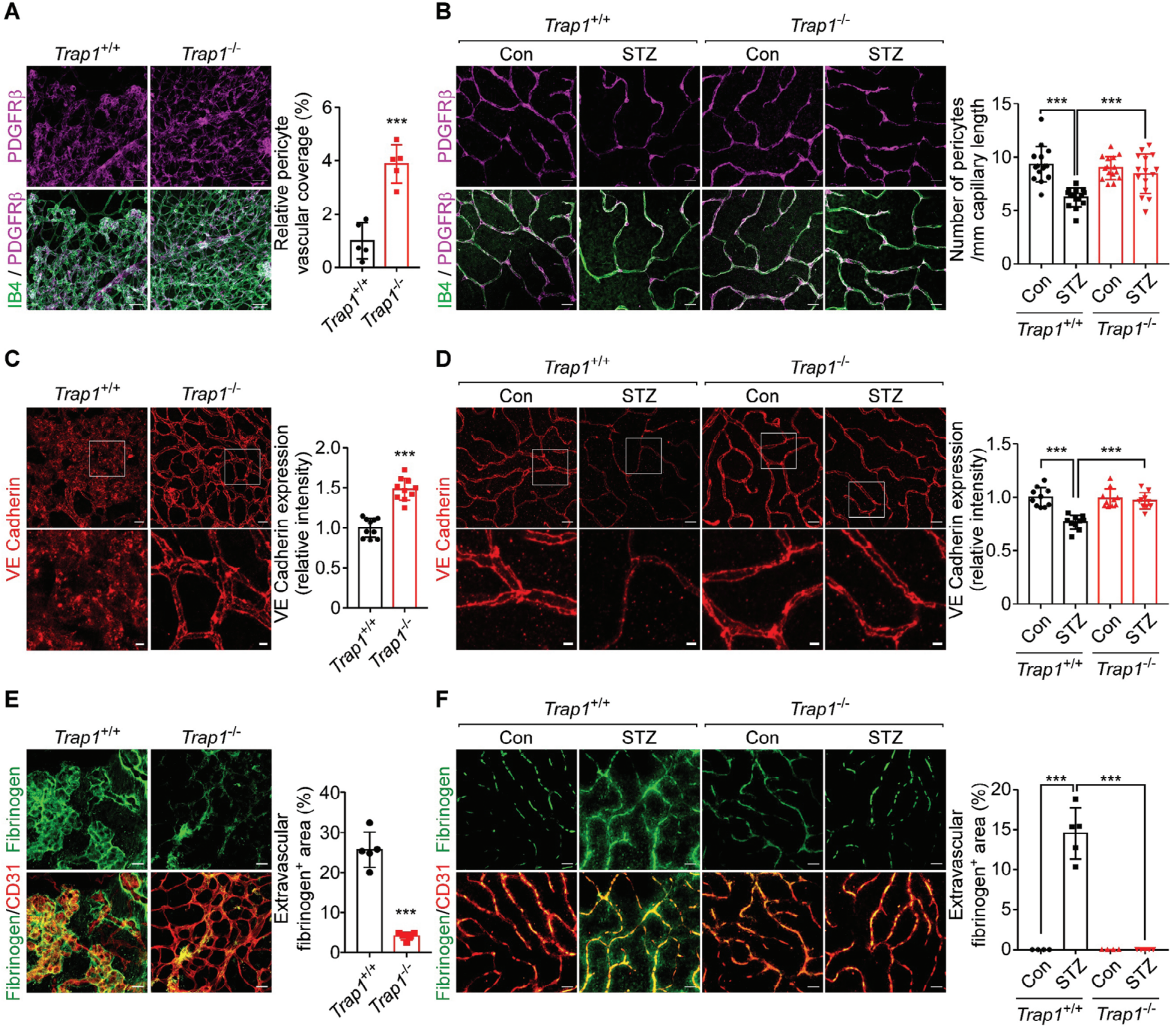
TRAP1 regulation of vascular organization and permeability. A) Pericyte vascular coverage in P17 OIR mouse retinas. Left. Whole‐mount retinas collected from *Trap1* ^+/+^ and *Trap1* ^−/−^ P17 OIR mice were stained with an anti‐PDGFR‐β antibody (magenta) and IB4 (green) to label PCs and ECs, respectively, and analyzed by confocal microscopy. Scale bar, 50 µm. Right. The ratio of the PDGFR^+^ area to the IB4^+^ area was calculated to measure pericyte coverage in vascularized areas (*n* = 5 mice/group). Neovascular tufts were not included in analysis. B) Pericyte coverage in STZ mouse retinas. Left. Whole‐mount retinas collected from STZ mice were analyzed by confocal microscopy as in (A). Scale bar, 20 µm. Right. The number of PCs per millimeter of capillary length was counted (*n* = 15; 5 mice/group, 3 fields/mouse). C) Endothelial junction integrity in P17 OIR mouse retinas. Left. Whole‐mount retinas collected from *Trap1* ^+/+^ and *Trap1* ^−/−^ OIR mice were stained with an anti‐VE‐cadherin antibody to visualize adherens junctions. Scale bars, 20 µm (top) and 5 µm (bottom). Right. The intensities of VE‐cadherin staining were calculated and compared (*n* = 10; 4 mice/group, 2–3 fields/mouse). D) Endothelial junction integrity in STZ mouse retinas. Left. Whole‐mount retinas collected from STZ mice were analyzed by confocal microscopy as in (C). Scale bar, 20 µm (top) and 5 µm (bottom). Right. Quantification of VE‐cadherin staining intensity (*n* = 10; 4 mice/group, 2–3 fields/mouse). E) Vascular leakage in P17 OIR mouse retinas. Left. Whole‐mount retinas collected from *Trap1* ^+/+^ and *Trap1* ^−/−^ OIR mice were stained with anti‐fibrinogen (green) and anti‐CD31 (red) antibodies. Scale bars, 50 µm. Right. Quantification of fibrinogen signals (*n* = 5 mice/group, 1 field/mouse). F) Vascular leakage in STZ mouse retinas. Left. Whole‐mount retinas collected from STZ mice were analyzed by confocal microscope as in (E). Scale bars, 10 µm. Right. Quantification of fibrinogen signal (*n* = 4–5; 4 mice/group, 1–2 fields/mouse). Data information: Data are expressed as mean ± SEM. Student *t*‐test, ^***^ *P* < 0.001.

Among HIF1α‐regulated cytokines, angiopoietin‐2 (ANG2) promotes the pathologic angiogenic functions of VEGF, and is implicated in the detachment and loss of pericytes (PCs) from endothelial cells (ECs), leading to BRB breakdown and increased vascular permeability.^[^ ^21^ ^]^ These histopathologic features were observed in both OIR and STZ wild‐type mice (**Figure** **4**A–F). Expression of ANG2 was elevated in OIR and STZ mouse retinas, and was significantly reduced in *Trap1* ^−/−^ mice at both the mRNA and protein levels; however, ANG1 expression was unaffected by *Trap1* ablation in OIR and STZ mice (Figure S9A–F, Supporting Information). Consequently, in *Trap1* ^−/−^ OIR and STZ mice, the ratio of PCs to ECs and expression of the adherens junction protein VE‐cadherin were restored (Figure 4A–D), indicating that TRAP1 inhibition restored PC‐EC integrity. Restoration of BRB integrity was further demonstrated by decreased extravasations of fibrinogen exudates and red blood cells (Figure 4E,F; Figure S10A,B, Supporting Information). Similarly, fluorescein angiography and fluorescein‐dextran permeability analyses revealed that the elevated retinal vascular leakages observed in *Trap1* ^+/+^ STZ mice were reduced in *Trap1* ^−/−^ mice (Figure S11A,B, Supporting Information).

To further examine the PC‐EC interaction, cocultures of human umbilical vein endothelial cells (HUVECs) and human brain vascular pericytes (HBVPs) were treated with conditioned media (CM) collected from MIO‐M1 cells exposed to hypoxia (Figure S12A, Supporting Information). CM obtained from cells treated with TRAP1‐targeting siRNA reduced tube formation areas and increased the PC‐EC ratio compared with CM obtained from cells treated with control siRNA (Figure S12B–D, Supporting Information). Similarly, treatment with CM obtained from MIO‐M1 cells depleted of ANG2 or HIF1α using siRNAs reduced tube formation areas and elevated the PC‐EC ratio (Figure S12E–G, Supporting Information). Collectively, these results indicate that TRAP1 inhibition not only suppresses retinal neovascularization but also restores the BRB in mice with ischemic retinopathy, potentially by decreasing ANG2 expression.

Depletion of TRAP1 using siRNAs resulted in decreases in the levels of various angiogenic factors released from hypoxic Müller cells. The angiogenic factors included monocyte chemoattractant protein‐1 (MCP‐1), granulocyte macrophage colony‐stimulating factor (GM‐CSF), heparin‐binding EGF‐like growth factor (HB‐EGF), chemokine ligand 16 (CXCL16), interleukin‐8 (IL‐8), and pentraxin 3 (PTX‐3) (Figure S13A–C, Supporting Information). Since these factors are also increased in DR patients and implicated in the pathogenesis of retinopathy,^[^ ^22^ ^]^ the reduced retinal pathogenesis observed upon TRAP1 inhibition might be associated with decreases in the levels of these factors, as well as those of VEGF, ANGPTL4, and ANG2.

### 2.5 TRAP1 Inhibition Activates Calpain‐1 to Trigger HIF1α Proteolysis

TRAP1 elevates the cellular concentration of succinate, leading to inhibition of PHD and subsequent stabilization of HIF1α, by inactivating succinate dehydrogenase (SDH) in cancer cells.^[^ ^23^ ^]^ However, TRAP1‐HIF1α regulation in the retina does not involve SDH and PHD because it was unaffected by treatment with a large excess of succinate (Figure S14A, Supporting Information), the PHD inhibitor dimethyloxalylglycine (DMOG), and a HIF1α mutant (P402A and P564A) lacking PHD hydroxylation sites (Figure S14B, Supporting Information). 17‐Dimethyl‐aminothylamino‐17‐demethoxy‐geldanamycin (DMAG), which inhibits cytoplasmic, but not mitochondrial Hsp90s,^[^ ^24^ ^]^ did not affect the stability of HIF1α, indicating that the regulatory mechanism is mitochondria‐specific,^[^ ^25^ ^]^ (Figure S14C, Supporting Information).

Interestingly, the proteasome inhibitor MG132 completely blocked HIF1α degradation triggered by TRAP1 inhibition, but lactacystin did not (Figure S14D,E, Supporting Information). Prior studies have reported that calpains, which are calcium‐activated proteases, are inhibited by MG132 but not by lactacystin,^[^ ^26^ ^]^ and can proteolyze HIF1α independently of PHD.^[^ ^27^ ^]^ Further, TRAP1 inhibition increases mitochondrial calcium discharge by increasing cyclophilin D (CypD) activity to trigger opening of the mitochondrial permeability transition pore (mPTP).^[^ ^3^, ^4^, ^28^ ^]^ Together, these data suggest that TRAP1 regulation of HIF1α is mediated by calcium and calpains.

**Figure 5:**
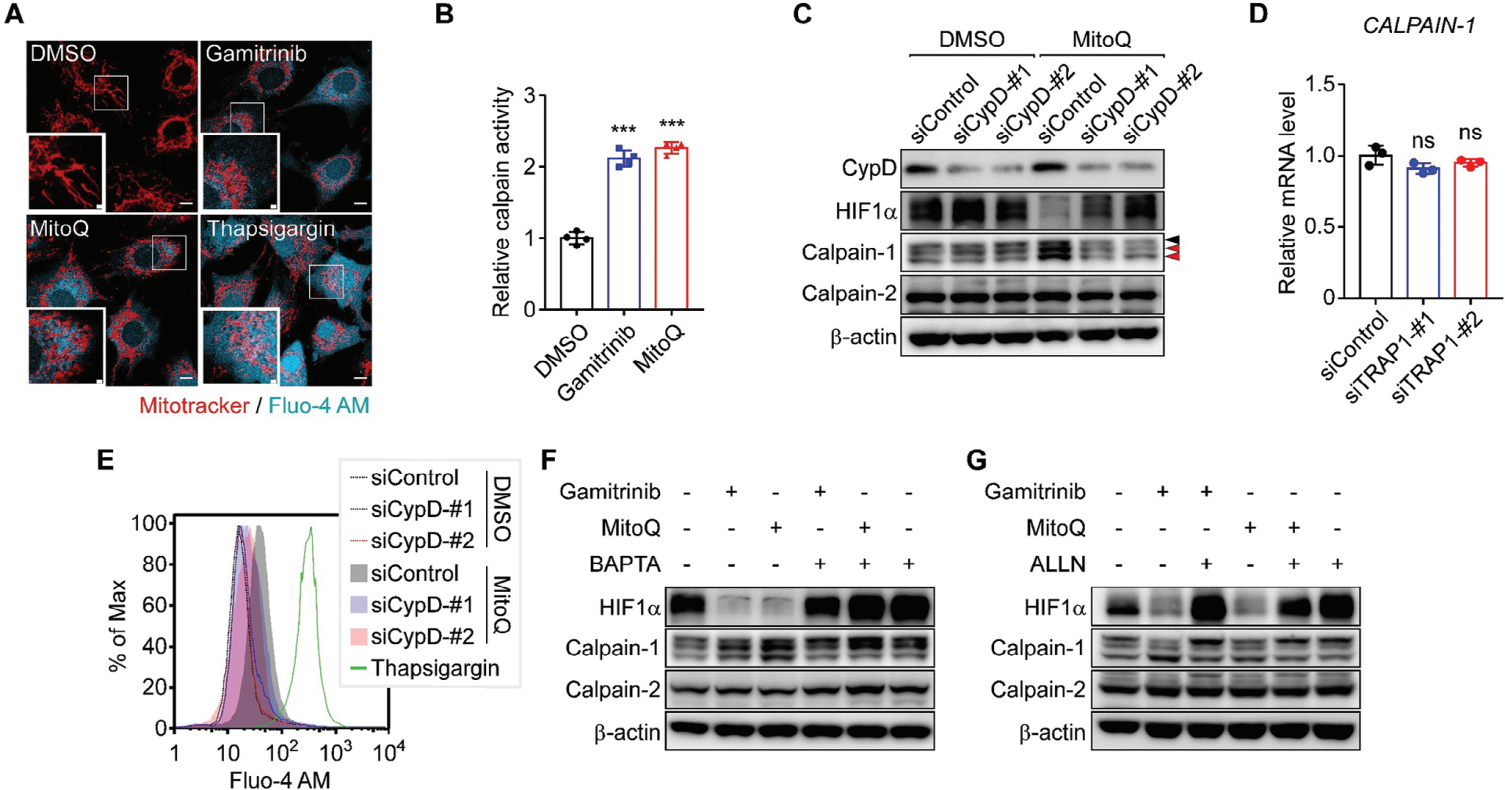
TRAP1 inhibition induces mPTP opening, mitochondrial calcium discharge, and calpain‐1 activation. A) Visualization of cytoplasmic calcium. Fluo‐4 AM‐labeled MIO‐M1 cells were incubated with the TRAP1 inhibitors gamitrinib and MitoQ^[^ ^32^, ^41^ ^]^ for 6 h or thapsigargin for 30 min under hypoxia, and analyzed by confocal microscopy. Scale bars, 10 µm and 2 µm (inset). B) Calpain activation by TRAP1 inhibitors. MIO‐M1 cells were treated with a TRAP1 inhibitor, 3 µM gamitrinib or 0.5 µm MitoQ, for 6 h under hypoxia (*n* = 4). Enzyme activity was measured using a fluorogenic calpain substrate as described in the Experimental Section. C) Restored HIF1α expression upon CypD inhibition. MIO‐M1 cells were incubated with control or CypD‐targeting siRNAs, treated with the TRAP1 inhibitor MitoQ for 6 h under hypoxia, harvested, and analyzed by western blotting. Black and red arrows indicate pro and autolyzed forms of calpain‐1, respectively. D) *Calpain‐1* mRNA expression upon TRAP1 depletion. MIO‐M1 cells were incubated with TRAP1‐targeting siRNAs for 48 h, exposed to hypoxia for 6 h, harvested, and analyzed by qPCR (*n* = 4). E) Modestly elevated cytosolic calcium by TRAP1 inhibition. After siRNA knockdown of CypD, Fluo‐4 AM‐labeled MIO‐M1 cells were incubated under hypoxic conditions with MitoQ for 6 h. Cells were then analyzed by flow cytometry to detect cytoplasmic calcium. F) Inhibition of HIF1α degradation by calcium chelation. MIO‐M1 cells were incubated with TRAP1 inhibitors, 3 µm gamitrinib, and 0.5 µM MitoQ, and a cell‐permeable calcium chelator, BAPTA (10 µM), for 6 h as indicated under hypoxia and analyzed by western blotting. G) Blocked HIF1α degradation by calpain inhibition. MIO‐M1 cells under hypoxia were incubated with 3 µm gamitrinib, 0.5 µm MitoQ, and 10 µm ALLN (calpain inhibitor) as indicated for 6 h and analyzed by western blotting. Data information: Data are expressed as mean ± SEM. Student *t*‐test, ^***^ *P* < 0.001; ns, not significant.

TRAP1 inhibition elevated the cytoplasmic calcium concentration and calpain enzyme activity (**Figure** **5**A,B). Subsequently, among the ubiquitously expressed isoforms, calpain‐1, but not calpain‐2, was activated as indicated by calpain autolysis (Figure 5C).^[^ ^29^ ^]^ However, the expression of calpain‐1 was unaffected (Figure 5D). Previous reports suggest that calpain‐1 is activated in the micromolar range of calcium concentration and calpain‐2 near the millimolar range.^[^ ^30^ ^]^ Consistently, compared with the substantial calcium release triggered by thapsigargin from the ER, TRAP1 inhibition only modestly increased cytoplasmic calcium levels (Figure 5A,E), which was fully reversed by inhibition of CypD by siRNAs (Figure 5C,E), indicating the involvement of mitochondrial calcium discharge via the mPTP in HIF1α degradation.

**Figure 6:**
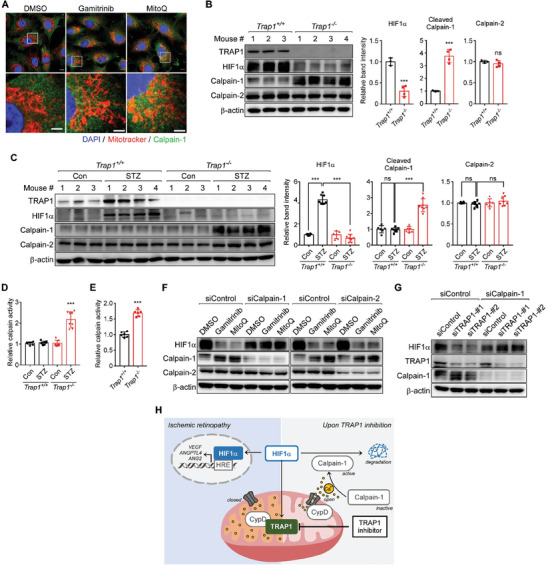
TRAP1 inhibition triggers calcium/calpain‐1‐dependent HIF1α degradation. A) Staining of mitochondria and calpain‐1. MitoTracker‐labeled MIO‐M1 cells were exposed to 3 µm gamitrinib or 0.5 µm MitoQ for 6 h under hypoxia and analyzed by immunocytochemistry with an anti‐calpain‐1 antibody. Scale bars, 10 µm (top) and 5 µm (bottom). B) Calpain autolysis in OIR mouse retinas. Left. Retinas collected from *Trap1* ^+/+^ (n = 3 mice) and *Trap1* ^−/−^ (*n* = 4 mice) OIR mice were analyzed by western blotting. Right. Protein band intensities of HIF1α, cleaved calpain‐1, and calpain‐2 were normalized to those of β‐actin and compared. C) Calpain autolysis in STZ mouse retinas. Left. Retinal samples collected from STZ (*n* = 4 mice) and age‐matched control (*n* = 3 mice) mice with the *Trap1* ^+/+^ or *Trap1* ^−/−^ genotype were analyzed by western blotting. Right. Protein band intensities were analyzed as in (B). D,E) Calpain activity in mouse retinas. Calpain enzyme activities were analyzed in retinas collected from *Trap1* ^+/+^ and *Trap1* ^−/−^ STZ (D, *n* = 8 mice/group) and OIR (E, *n* = 6 mice/group) mice and compared. F) Depletion of calpains by siRNAs. Calpain‐1‐ and calpain‐2‐targeting siRNA‐treated MIO‐M1 cells were incubated with 3 µm gamitrinib or 0.5 µm MitoQ for 6 h under hypoxia and analyzed by western blotting. G) Depletion of calpain‐1 and TRAP1 by siRNAs. MIO‐M1 cells treated with siRNAs as indicated were exposed to hypoxia for 6 h and analyzed by western blotting. H) HIF1α degradation following TRAP1 inhibition. TRAP1 inhibition caused mild mitochondrial calcium discharge into the cytoplasm by opening CypD‐regulated mPTPs. Subsequent activation of calpain‐1 proteolytically degrades HIF1α. Data information: Data are expressed as mean ± SEM. Student *t*‐test, ^***^ *P* < 0.001; ns, not significant.

Both HIF1α degradation and calpain‐1 activation were fully reversed by treatment with CypD‐targeting siRNAs, a calcium chelator (1,2‐bis (o‐aminophenoxy) ethane‐*N*, *N*, *N*', *N*'‐tetraacetic acid (BAPTA)), and a calpain inhibitor (ALLN) (Figure 5C,F,G), further confirming that the mechanism is dependent on mitochondrial calcium/calpain‐1. TRAP1 inhibition increased calpain‐1 staining in mitochondria‐enriched perinuclear regions (**Figure** **6**A), further supporting the notion that calpain‐1 was activated by the discharge of mitochondrial calcium. Similarly, the active (cleaved) forms of calpain‐1 were elevated in OIR and STZ retinas isolated from *TRAP1* ^−/−^ mice, but not in control retinas or OIR and STZ retinas isolated from *Trap1* ^+/+^ mice (Figure 6B,C). Proteolytic enzyme activity of calpain was also elevated in OIR and STZ retinas isolated from *Trap1* ^−/−^ mice, but not in control retinas or OIR and STZ retinas isolated from *Trap1* ^+/+^ mice (Figure 6D,E). Finally, siRNA‐mediated depletion of calpain‐1 abolished HIF1α degradation in MIO‐M1 cells, but treatment with calpain‐2‐targeting siRNA did not (Figure 6F,G), confirming the pathway is calpain‐1‐dependent. These data indicate that TRAP1 inhibition activates calpain‐1 to destabilize HIF1α by inducing mild mitochondrial calcium release into the cytosol (Figure 6H).

Given that TRAP1 is implicated in the generation of mitochondrial reactive oxygen species (ROS),^[^ ^24b^ ^]^ resulting in the stabilization of HIF1α,^[^ ^31^ ^]^ we analyzed retinal ROS production using dihydroethidium (DHE) staining. The levels of DHE staining were comparable between *Trap1* ^+/+^ and *Trap1* ^−/−^ mouse retinas from both STZ and OIR models (Figure S15A,B, Supporting Information), indicating the retinal ROS production is not affected by TRAP1 expression.

### 2.6 Pharmacological Inhibition of TRAP1 Alleviates Vascular Pathologies in OIR Mice

**Figure 7:**
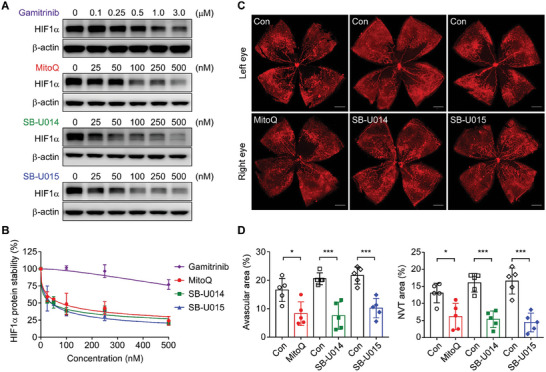
Normalized retinal vascularization upon topical application of TRAP1 inhibitors. A) HIF1α degradation induced by TRAP1 inhibitors. MIO‐M1 cells were incubated with various concentrations of TRAP1 inhibitors as indicated for 6 h under hypoxia and analyzed by western blotting. B) Quantification of HIF1α protein degradation. The HIF1α band intensities in western blot data obtained as in (A) were analyzed and compared. The data are mean ± SEM from four independent experiments. C) Vascular structure of P17 OIR mouse retinas after topical drug application. Vehicle and 2 mM TRAP1 inhibitors were topically administered to the left and right eyes, respectively, of OIR mice at P12 once per day for 5 days. At P17, mice were sacrificed and analyzed by whole‐mount staining with an anti‐CD31 antibody and confocal microscopy. Scale bar, 500 µm. D) Quantification of avascular areas and neovascular tuft. Retinal images collected as in (C) were quantitatively analyzed (*n* = 5 mice/group). Data information: Data are expressed as mean ± SEM. Student *t*‐test, ^***^ *P* < 0.001; ^*^ *P* <0.5.

Among representative allosteric and orthosteric TRAP1 inhibitors,^[^ ^24^, ^32^ ^]^ MitoQ was more than 10‐fold higher potency than gamitrinib in inducing the degradation of HIF1α (**Figure** **7**A,B). Treatment with MitoQ or gamitrinib did not affect HIF1α expression, the cytosolic calcium concentration, or calpain‐1 activation in *Trap1* ^−/−^ cells (Figure S16A–C, Supporting Information), indicating that it induces HIF1α degradation in a TRAP1‐specific manner. Similar to the phenotype of *Trap1* KO mice, intravitreal injection of MitoQ dramatically reduced both avascular and neovascular tuft areas in OIR mice (Figure S17A,B, Supporting Information). Furthermore, topically applied MitoQ showed potent activity, and its effects were comparable with those observed in *Trap1* KO mice (Figure S17C,D, Supporting Information), indicating that MitoQ was efficiently delivered to the posterior segment of the eye.

TRAP1 inhibitors derived from MitoQ, SB‐U014, and SB‐U015, are more effective in inhibiting TRAP1 than MitoQ and lack antioxidant activity.^[^ ^32^ ^]^ These derivatives induced HIF1α degradation more strongly than MitoQ (Figure 7A,B). The IC_50_ values of SB‐U014, SB‐U015, and MitoQ were 0.040, 0.041, and 0.077 µM, respectively. SB‐U014, SB‐U015, and MitoQ elicited cytotoxic effects on mouse primary Müller cells at concentrations of 27, 32, and 22 µM, respectively; therefore, there were several hundred‐fold (295–773‐fold) differences between the therapeutic and toxic doses (**Table** **1**; Figure S18A, Supporting Information). This indicates that the safety margins of all these drugs are very high, and that of SB‐U015 is highest. Topically applied MitoQ, SB‐U014, and SB‐U015 reduced the extent of avascular and neovascular tuft areas in OIR mice (Figure 7C,D).

**Table 1:** Therapeutic index.

Drug	HIF1α degradation^a)^ (IC_50_, µM)	Normal cell cytotoxicity^b)^ (IC_50_, µM)	Therapeutic index (B/A)
MitoQ	0.077 ± 0.016	22.47 ± 0.46	295.81
SB‐U014	0.040 ± 0.091	27.47 ± 0.77	694.92
SB‐U015	0.041 ± 0.083	31.78 ± 0.76	773.05

^a)^ IC_50_ values of HIF1α protein degradation in MIO‐M1 cells upon TRAP1 inhibitor treatment were analyzed as in Figure 7A,B (*n* = 4);

^b)^ IC_50_ values of the cytotoxic activities of the drugs in primary Müller cells were calculated as in Figure S18A, Supporting information (*n* = 4). The therapeutic index^[^ ^42^ ^]^ was determined as (IC_50_ for normal cell cytotoxicity)/(IC_50_ for HIF1α degradation). Data information: Data are expressed as mean ± SEM.

Following topical administration of SB‐U015 to mice and rabbits, the peak drug concentration (C_max_) was reached at 0.83 and 1.5 h (T_max_), respectively. The C_max_ and area under the concentration‐time curve to last measurement (AUC_last_) values were 1.8‐ and 2‐fold higher in rabbit retina than in P12 mouse retina, respectively (**Table** **2**). These data suggest that topically administered SB‐U015 can be sufficiently delivered to the retina irrespective of ocular size and anatomy. Furthermore, all drugs were nonirritants in the reconstructed human cornea epithelial model according to the OECD guidelines for test chemicals (Figure S18B and Table S1, Supporting Information), and there was no noticeable histologic abnormality after topical application of TRAP1 inhibitors based on hematoxylin and eosin (H&E) staining (Figure S18C, Supporting Information), suggesting that topical application of the drugs is safe.

**Table 2:** Ocular pharmacokinetics analysis after topical administration of SB‐U015.

	Mouse	Rabbit				
	Retina	Retina	Sclera	Aqueous humor	Cornea	Plasma
C_max_ [ng/g]	36.5 ± 7.2	65.6 ± 20.1	664.3 ± 122.8	NA	3272.4 ± 283.7	NA
AUC_last_ [hng/g]	44.8 ± 6.2	90.9 ± 16.9	1697.9 ± 318.7	NA	9226.6 ± 637.6	NA
T_max_ [h]	0.8 ± 0.2	1.5 ± 0.8	1.1 ± 0.3	NA	2.6 ± 0.9	NA

*NA: Not applicable because the analyte was not detected.

Data information: Data are expressed as mean ± SEM (n = 4) except for plasma (n = 2) and mouse retina (n = 3).

Collectively, these data demonstrate that TRAP1 inhibitors can be developed as therapeutic agents to treat ischemic retinopathy, alleviating pathological angiogenesis and restoring the normal vascular architecture via topical application.

## 3 Discussion

HIF1α is a transcription factor that contributes to the pathogenesis of ischemic retinopathy by up‐regulating expression of proangiogenic factors.^[^ ^33^ ^]^ The present study demonstrated that mitochondrial chaperone TRAP1 is necessary for HIF1α‐dependent retinal pathogenesis in mouse models of ischemic retinopathy. Genetic and pharmacologic inhibition of TRAP1 triggered calpain‐1 activation and subsequent HIF1α degradation in both ischemic OIR and diabetic STZ mice, which not only suppressed pathologic neovascularization but also restored BRB integrity and microvascular structure without any recognizable adverse effects. Our data collectively indicate that TRAP1 is a novel druggable target to treat HIF1α‐driven ischemic retinopathies, and that small molecule inhibitors of TRAP1, such as MitoQ and SB‐U015, could be utilized to develop potent therapeutics with well‐tolerated topical administration.

In the ischemic retina, TRAP1 altered mitochondrial signaling by inhibiting CypD, the master regulator of mPTPs, and subsequently decreasing mPTP opening.^[^ ^3^, ^4^ ^]^ Although the molecular components are controversial, there are at least two types of mPTPs, namely, a reversible low conductance pore with physiological functions and an irreversible high conductance pore involved in cell death.^[^ ^34^ ^]^ In many cancer cells, TRAP1 inhibition triggers opening of high conductance pores by activating CypD, which results in massive calcium discharge, leading to mitochondrial dysfunction and cell death.^[^ ^3^, ^4^ ^]^ However, in retinal cells, TRAP1 inhibition seems to trigger opening of the low conductance mPTP and a small amount of mitochondrial calcium discharge because it does not elicit cytotoxic effects; activates calpain‐1, which requires a low micromolar concentration of calcium; and does not activate calpain‐2, which requires a high micromolar to millimolar concentration of calcium.^[^ ^29^ ^]^ Due to the diffusive nature and relatively low level of calcium released upon TRAP1 inhibition, calpain‐1 was elevated (and probably activated) primarily near to mitochondria. This is reminiscent of the recruitment of calpains to the endoplasmic reticulum and Golgi where the local calcium concentration is elevated for their efficient activation.^[^ ^35^ ^]^ Collectively, our data suggest that spatiotemporal regulation of calpain‐1 is coordinated by mPTP opening and mitochondrial calcium discharge in the hypoxic retina. Thus, in diseased hypoxic retinal cells, expression of TRAP1 is induced to close mPTPs and maintain mitochondrial calcium storage, which is essential for activation of HIF1α and development of retinopathy.

To induce OIR, mouse pups are reared in a hyperoxic chamber (P7‐P12, 75% O_2_), which mimics oxygen exposure in preterm infants in neonatal intensive care.^[^ ^7^, ^13^ ^]^ Thus, the OIR model has been widely used to study ROP and develop therapeutic interventions,^[^ ^13a^ ^]^ suggesting that TRAP1 inhibitors could be used to develop therapeutics for ROP acting via novel mechanisms. In addition, OIR mimics pathologic retinal neovascularization that is driven by hypoxia and aberrant expression of angiogenic factors in proliferative DR .^[^ ^9^ ^]^ Mice with STZ‐induced diabetes exhibited retinal hypoxia and HIF1α activation similar to those observed in OIR mice, which likely contributed to early vascular pathologies characteristic of non‐proliferative DR, such as vascular hyperpermeability and capillary degeneration.^[^ ^7a^ ^]^ Therefore, further research into the functions of TRAP1 in the progression of DR could lead to the development of novel treatments for early and advanced stages of DR based on TRAP1 inhibitors.

Laser photocoagulation surgery, which permanently destroys peripheral retinal vessels, is currently the standard of care for severe ROP in preterm infants.^[^ ^7c^ ^]^ Intravitreal injection of anti‐VEGF drugs has recently been combined with laser photocoagulation therapy to improve the treatment outcome by minimizing irreversible loss of the visual field due to peripheral retinal ablation.^[^ ^36^ ^]^ However, because VEGF plays an important role in vascular development of the retina and other tissues in preterm infants, the potential ocular and systemic side effects of intravitreally administered anti‐VEGF drugs cannot be ignored.^[^ ^11^, ^37^ ^]^ Contrastingly, TRAP1 inhibition is unlikely to pose a risk for these side effects, as expression of angiogenic factors, including VEGF, ANGPTL4, and ANG2, was only suppressed partially to physiological levels in OIR mice. Consistently, vascular regression, which can be caused by complete inhibition of angiogenic factors, was not observed in *Trap1* ^−/−^ mice. This finding indicates that TRAP1 did not affect basal expression of angiogenic factors during physiologic retinal vascular development. Collectively, TRAP1 inhibition suppresses only aberrant angiogenic factors produced by stabilized HIF1α in the diseased ischemic retina. This intervention can thus enable normalization of vascular structure by restoring balanced expression of angiogenic factors in OIR mice. Thus, TRAP1 should be further evaluated as a novel therapeutic target for development of safe and effective ROP interventions that do not present the concerns of vascular disruption induced by laser surgery and VEGF inactivation.

Intravitreal injection of anti‐VEGF drugs has also been widely performed as a standard of care for diabetic retinopathies such as diabetic macular edema and PDR.^[^ ^7^, ^9^ ^]^ However, targeting VEGF alone has several limitations. Specifically, some patients are refractory to anti‐VEGF drugs,^[^ ^9^, ^38^ ^]^ and repeated therapy, especially of DR patients, can lead to tissue atrophy and ischemia.^[^ ^39^ ^]^ Drug irresponsiveness can arise, at least in part, due to other HIF1α‐inducible factors such as ANGPTL4 and ANG2,^[^ ^10^ ^]^ leading to neovascularization and BRB deterioration in the absence of VEGF. Consistently, combined neutralization of such proangiogenic factors has better efficacy than VEGF monotherapy in animal disease models,^[^ ^40^ ^]^ and a bispecific antibody drug, faricimab, has recently been approved for the treatment of ocular diseases.^[^ ^21b^ ^]^ These considerations support the proposition that a TRAP1 inhibition strategy could be effective for the treatment of ischemic retinopathies, which would inactivate the upstream master regulator HIF1α to simultaneously block pathological up‐regulation of multiple angiogenic regulators.

Currently, no ophthalmic preparations are available for the treatment of retinal diseases, which is likely due to poor drug delivery to the posterior segment. Thus, more research will be required to understand the factors affecting the efficiency of drug delivery to the retina to develop optimal and effective ophthalmic solutions. To this end, in this study, we compared the ocular pharmacokinetics of the most potent TRAP1 inhibitor SB‐U015 in mice and rabbits. The results revealed that rabbits accumulated more SB‐U015 in their retinas than mice after topical administration, indicating that the drug can reach the retina irrespective of eyeball size and anatomy. These findings suggest that eye drop application of TRAP1 inhibitors has the potential to achieve effective drug concentrations in human retinas, providing a noninvasive treatment option for ischemic retinopathies.

In this study, the mitochondrial chaperone TRAP1 was identified and validated as a target protein for development of effective therapeutics for ischemic retinopathies with a novel mode of action. Furthermore, considering that small molecules have high tissue penetration and improved stability compared with large molecular weight antibody drugs, TRAP1 inhibitors could be not only formulated as noninvasive ophthalmic solutions as shown here, but also combined with various drug delivery systems such as drug‐loaded contact lens and long‐lasting implants to avoid or reduce patient treatment burdens associated with repeated intravitreal injections.

## 4 Experimental Section

### Chemicals and Antibodies

All chemicals were purchased from Sigma (Burlington, MA, USA) unless indicated otherwise. DMOG, ALLN (calpain inhibitor), lactacystin, and thapsigargin were purchased from Cayman (Ann Arbor, MI, USA). MitoQ and 17‐DMAG were purchased from MedChemExpress (Monmouth Junction, NJ, USA) and LC laboratories (Woburn, MA, USA), respectively. Antibodies against TRAP1 (#612344), HIF1α (#610958), CD31 (#550274), VE‐cadherin (#555289), Ter119 (#561033), Hsp90 (#610418), and Hsp70 (#610607) were purchased from BD Biosciences (San Jose, CA, USA). Isolectin B4 (IB4) conjugated with Alexa Fluor 488 (#I21411) and antibodies against TRAP1 (#PA5‐27596), calpain‐1 (#MA3‐940), and PDGFR‐β (#14‐1402‐82) were purchased from Invitrogen (Waltham, MA, USA). Antibodies against HIF1α (#NB 100–479), and VEGF (#NB 100–664) were purchased from Novus Biologicals (Centennial, CO, USA). Anti‐collagen IV (#ab19808), anti‐TRAP1 (#ab151239), anti‐F4/80 (#ab6640), and anti‐GFAP (ab#53554) antibodies were purchased from Abcam (Cambridge, UK). Antibodies against calpain‐2 (#2539) and cleaved caspase‐3 (#9664) were purchased from Cell Signaling Technology (Danvers, MA, USA). Antibodies against β‐actin (#MP 691001) and glutamine synthetase (#MAB302) were purchased from Millipore (Burlington, MA, USA). Antibodies against ANG2 (#MAB 098–100) and VEGF (#AF‐493‐SP) were purchased from R&D Systems (Minneapolis, MN, USA). Antibodies against ANGPTL4 (#LS‐C331822‐100; Lifespan Bioscience; Seattle, WA, USA), Chk1 (#sc‐8408; Santa Cruz; Dallas, TX, USA), CypD (#AP1035; Calbiochem; San Diego, CA, USA), and fibrinogen (#F8512; Sigma) were obtained from the indicated suppliers.

### Animal Models of Retinopathy

All animal experiments except ocular pharmacokinetic analyses in rabbits were approved by the Institutional Animal Care and Use Committee (IACUC) of the Ulsan National Institution of Science and Technology (IACUC‐UNIST20‐01, IACUC‐UNIST21‐21, and IACUC‐UNIST21‐30). The rabbit ocular pharmacokinetic experiment was approved by the IACUC of Knotus (KNOTUS IACUC 22‐KE‐0109) and conducted in Knotus (Republic of Korea). Animals were housed in a room with constant humidity (40–60%) and temperature (20–25°C) under a 12 h light‐dark cycle. Mice were given free access to food and water.

To induce OIR (Figure 1A), mouse pups were exposed to 75% oxygen at postnatal day 7 (P7) until P12 together with their mothers in a hyperoxic chamber (InVivo Cabinet Model 15; Coy Laboratory; Grass Lake, MI, USA). Hyperoxia‐exposed pups were returned to room air at P12 with nursing mothers until P17. At P17, mice were sacrificed, and eyes were enucleated.

*Trap1* ^−/−^ mice were designed and generated by the UC Davis Mouse Biology Program. The *Trap1* locus was genotyped using the following primers: 5′‐GAGGAGTGGTATTGGAAGTATGGAC‐3′ and 5′‐AGTAGCTTTCCCTTATATACAGAATGCC‐3′ for the wild‐type allele, and 5′‐AGTTCAATGCAAGACTTCTGCCAAGG‐3′ and 5′‐GAGATGGCGCAACGCAATTAAT‐3′ for the *Trap1* KO allele (Figure S2A, Supporting Information).

For the STZ mouse model of type 1 diabetes (Figure S6A, Supporting Information), STZ (75 mg/kg/day) was intraperitoneally injected into 8‐week‐old male C57BL/6J mice for 5 days. The fasting blood glucose levels of mice were measured every week with a glucometer to monitor development and maintenance of diabetes. Mice with glucose levels of 350 mg dl^−1^ or higher were deemed diabetic. Mouse eyes were collected and analyzed at 16 weeks after STZ treatment.

### Intravitreal Injection and Topical Treatment of Drugs in OIR Mice

For intravitreal injection, MitoQ was injected into the vitreous cavity using a Nanoliter 2000 microinjector (World Precision Instruments; Sarasota, FL, USA) fitted with glass capillary pipettes under anesthesia. In total, 1 µl MitoQ (0.1 mg/ml) was delivered into the right eye of an OIR mouse once at P12. The contralateral eye was injected with vehicle (0.1% DMSO prepared in distilled water) as a control. For topical treatment, 10 µl drug dissolved in 80% Liposic (Bauch Health Companies Inc.; Laval, Canada) was applied to the right eye, and vehicle was applied to the contralateral left eye for 1 min, and then residual drugs were wiped out. Mice were treated with the drugs once or thrice daily from P12 to P17.

### Immunohistochemistry and Hypoxyprobe Staining

For hypoxic tissue staining, 60 mg/kg Hypoxyprobe (Burlington, MA, USA) was intravenously and intraperitoneally injected into STZ and OIR mice, respectively. After 90 min, mice were intracardially perfused with phosphate‐buffered saline (PBS), and eyes were harvested and fixed with 4% paraformaldehyde (PFA) overnight at 4°C. STZ mouse eyes underwent slide staining after production of paraffin blocks, and OIR mouse eyes underwent whole‐mount staining. Paraffin‐embedded tissue sections were analyzed by immunohistochemistry using a Rabbit and Mouse Specific HRP/DAB (ABC) Detection IHC Kit (Abcam) according to the manufacturer's instructions. Paraffin sections were dewaxed, rehydrated, and treated with the hydrogen peroxide block for 10 min, followed by heat‐induced antigen retrieval in 10 mm sodium citrate (pH 6.0) using a pressure cooker. Tissue slides were blocked with blocking solution followed by the Hypoxyprobe primary antibody overnight at 4 °C. The next day, slides were incubated with biotinylated goat antipolyvalent and streptavidin peroxidase, and then the DAB substrate reaction was performed. Tissue slides were scanned with a slide scanner microscope (Olympus; Tokyo, Japan) and analyzed using ImageJ.

### Fluorescein Angiography and Fluorescein‐Dextran Permeability Analyses

For angiographic analysis of retinal vascular leakage, 100 µl of 1% fluorescein sodium (Sigma) was intraperitoneally injected into age matched control and 16‐week‐old STZ mice, respectively. Mice were anesthetized with intraperitoneal injection of 2.5% avertin (250 mg/kg). Isopto Atropine drops (Alcon, Geneva, Switzerland) were used for dilation, followed by the application of GenTeal Tears (Alcon) for lubrication of mouse eyes. Retinal vessel leakage was imaged by an iVivo Funduscope (OcuScience, Henderson, NV, USA), and quantified using ImageJ.

For fluorescein‐dextran permeability analysis, mice were anesthetized with an intraperitoneal injection of 2.5% avertin (250 mg/kg), followed by the injection of 100 µl fluorescein‐dextran (1.25 mg/mouse, Sigma) into the left ventricle. After 5 min, eyes were collected, fixed in 4% PFA for 2 h on ice, and whole‐mounted onto glass slides after dissection. Fluorescence images were acquired using an LSM980 confocal microscope (Zeiss) and analyzed with ImageJ.

### Immunostaining of Whole‐Mount Retinas

Fixed eyes were dissected to isolate retinas under a microscope. Subsequently, flattened retinas were blocked, permeabilized, and exposed to primary and secondary antibodies as described for immunofluorescence staining. Retinas were immersed in mounting medium. Fluorescence images were acquired using an Axio Zoom fluorescence microscope (Zeiss; Oberkochen, Germany) or a LSM780 or LSM980 confocal microscope (Zeiss). Image analyses were performed with a Zeiss image analyzer.

### Immunofluorescence Staining

After heat‐induced antigen retrieval, tissue slides were permeabilized with PBS containing 1% Triton X‐100 for 1 h; blocked with PBS containing 5% fetal bovine serum (FBS), 5% bovine serum albumin (BSA), and 0.3% Triton X‐100 for 1 h at room temperature (RT); incubated with primary antibodies overnight at 4°C; and stained with Alexa Fluor‐labeled secondary antibodies (Invitrogen) for 1 h at RT. After incubation, the slides were stained with DAPI (Invitrogen) for 5 min at RT and immersed in mounting medium (Vector Laboratories; Newark, CA, USA). Images were acquired using a LSM780 confocal microscope (Zeiss). Image analyses were performed with Zeiss image analyzer.

### Dihydroethidium (DHE) Staining

After heat‐induced antigen retrieval, tissue slides were blocked with PBS containing 5% fetal bovine serum (FBS), 5% bovine serum albumin (BSA), and 0.3% Triton X‐100 for 1 h at room temperature (RT). After blocking, the slides were incubated with 5 µM DHE (Invitrogen) in 1XPBS solution for 30 min at 37 °C in dark, stained with DAPI (Invitrogen) for 5 min at RT, and then immersed in mounting medium (Vector Laboratories; Newark, CA, USA). 10 mm *N*‐acetyl cysteine (NAC; Sigma) was used to eliminate ROS as a negative control. Images were acquired using an LSM780 confocal microscope (Zeiss) and analyzed using a Zeiss image analyzer.

### Cell Culture and Knockdown Experiments

The human Müller cell line MIO‐M1 was a gift from Prof. S. Yoshida (University of Kurume, Kurume, Fukuoka, Japan), and was cultured in DMEM‐low glucose (Sigma) supplemented with 10% FBS (Gibco; Waltham, MA, USA) and 1% penicillin‐streptomycin (Gibco). GFP‐expressing HUVECs (Angio‐Proteomie; Boston, MA, USA) were cultured in EGM medium (Lonza; Basel, Switzerland). HBVPs (Sciencell; Carlsbad, CA, USA) were cultured in pericyte media (Sciencell). All cell lines were cultured as recommended by the manufacturer and incubated at 37 °C with 5% CO_2_. For knockdown experiments, siRNAs were transfected using G‐fectin (Genolution; Seoul, Republic of Korea) as instructed by the supplier. siRNAs targeting TRAP1, HIF1α, calpain‐1, calpain‐2, and CypD were synthesized by Genolution as follows:

**Table:** 

Species	Name	Sequence (5′→3′)
Mouse	siTRAP1‐#1	AAACATGAGTTCCAGGCAGAG
Mouse	siTRAP1‐#2	GCCCGTTCTCTGTACTCAGAA
Mouse	siHIF1α‐#1	GGGTTATGAGCCGGAAGAACT
Mouse	siHIF1α‐#2	GATGGAAGCACTAGACAAAGT
Human	siTRAP1‐#1	AAACATGAGTTCCAGGCCGAG
Human	siTRAP1‐#2	CCCGGTCCCTGTACTCAGAAA
Human	siCalpain‐1	GGAACAACGTGGACCCATA
Human	siCalpain‐2	CTATTGGCTTCGCGGTCTA
Human	siCypD‐#1	GGACTCTAATACCTGTTTA
Human	siCypD‐#2	GGCAGATGTCGTCCCAAAG
Human	siANG2	GTGACTGCCACGGTGAATAAT
Human	siHIF1α	GGCCACATTCACGTATATGAT

### Generation of *Trap1* Knockout (KO) Cell Lines

MIO‐M1 *Trap1* KO cell lines were generated using CRISPR/Cas9‐derived RNA‐guided endonucleases (Toolgen; Seoul, Republic of Korea). MIO‐M1 cells were co‐transfected with the Cas9 vector and TRAP1‐sgRNA expression vector (sgRNA sequence 1; 5′‐CTCGGCCTGGAACTCATGTTTGG‐3′ and sequence 2; 5′‐AGCTTTTGGACATTGTTGCCCGG‐3′) using jetPRIME transfection reagent (Polyplus; Illkirch‐Graffenstaden, France). Transfected cells were selected in culture medium containing 3 mg/ml puromycin (Sigma). Single‐cell colonies were collected using a cloning cylinder (I.D. × H 6.4 mm × 8 mm; Sigma) and analyzed using T7 endonuclease I to confirm *Trap1* KO.

### Flow Cytometric Analysis of Cellular Calcium

Cells (2 × 10^5^) were cultured on a 6‐well plate, treated with drugs under hypoxia or normoxia for 6 h, and stained with 2 µm Fluo‐4 AM (Invitrogen) for 30 min at RT. Fluorescence signals were detected using a flow cytometer (LSRFortessa; BD Biosciences).

### Isolation of Primary Müller Cells

Mouse primary Müller cells were isolated as reported previously.^[^ ^43^ ^]^ In brief, retinas were isolated from P5–P7 mice and dissociated using a Papain Dissociation Kit (Worthington Industries; Columbus, OH, USA). Collected retinal cells were incubated on a plate coated with 0.1% gelatin (Sigma), and the culture media (DMEM supplemented with 10% FBS and 1% penicillin‐streptomycin) was changed once every 2 days. Cells were subcultured into a new plate and incubated for another 7 days. Adherent cells were largely primary Müller cells, as confirmed by immunocytochemical analysis using an anti‐glutamine synthetase antibody (Millipore).

### Preparation of CM and the Tube Formation Assay

To collect CM from MIO‐M1 cells exposed to hypoxia, cells were transfected with siRNAs and incubated for 24 h in normoxia. The media were replaced by fresh DMEM/F12 supplemented with 1% FBS, and cells were incubated for another 24 h under normoxia or 1% O_2_ hypoxia chamber (SMA‐30D; Astec; Fukuoka, Japan). The media were collected, centrifuged at 130 g for 5 min, and stored at −80°C as CM (Figure S12A, Supporting information). In total, 8 × 10^4^ GFP‐expressing HUVECs and 1.6 × 10^4^ HBVPs labeled with CellTracker Red CMTPX (Invitrogen) were suspended in CM supplemented with 1% FBS and plated in a 35 mm coverglass‐bottom dish (Spl; Gyeonggi‐do, Republic of Korea) coated with Matrigel (Corning; Corning, NY, USA). Cells were incubated for 6 h to allow tube formation. Images were acquired using a LSM780 confocal microscope and analyzed using a Zeiss image analyzer.

### Proteome Profiler Human Angiogenesis Array

Proteome profiler arrays for 55 human angiogenesis‐related proteins were used according to the manufacturer's instructions (ARY007; Proteome Profiler Human Cytokine Array Kit, R&D Systems, Minneapolis, MN, USA). A total of 500 µl conditioned media (CM) collected from MIO‐M1 cells exposed to hypoxia was combined with biotinylated antibodies and incubated with the membrane overnight at 4 °C. On the following day, the membrane was washed with 1X wash buffer and subsequently incubated with Streptavidin‐HRP for 30 min. After incubation, the membrane was washed three times and developed using the ECL solution. Analysis was performed using a LAS4000 system (GE Healthcare; Chicago, IL, USA), and pixel density was quantified using ImageJ.

### Western Blotting

Cells and tissue samples were lysed using RIPA buffer (50 mM Tris, pH 8.0, 150 mm NaCl, 1% NP‐40, and 0.25% *N*‐deoxycholate) containing protease and phosphatase inhibitor cocktails (Roche). Lysates were separated by SDS‐PAGE and transferred to PVDF membranes. The membranes were blocked with 10% skim milk prepared in TBST (TBS (50 mM Tris and 150 mm NaCl, pH 7.6) containing 0.05% Tween‐20) for 1 h at RT and incubated with primary antibodies in antibody diluent solution (TBS containing 3 mm sodium azide and 0.1% BSA) overnight at 4 °C. After washing with TBST, the membranes were incubated with secondary antibodies for 1 h in 10% skim milk prepared in TBST. Finally, the membranes were washed three times with TBST, treated with ECL solution (Bio‐Rad; Hercules, CA, USA), and analyzed using a LAS4000 system (GE Healthcare; Chicago, IL, USA).

### Eye Irritation Test with MCTT HCE

An eye irritation test was performed using a 3D human corneal epithelial (HCE) model, called MCTT HCE (KeraSkin; Biosolution, Seoul, Republic of Korea), for OECD test guideline 492, following the manufacturer's instructions. In brief, HCE tissues were incubated overnight in medium provided by the supplier. Thereafter, 2 mm MitoQ, SB‐U014, and SB‐U015 were applied to the upper epithelial surface of HCE tissues and incubated for 10 min. After removing the drugs by washing the tissues with PBS, the tissues were further incubated for 16 h, and then subjected to the WST‐1 assay for viability assessment and fixed in 4% PFA for histological analysis.

### RNA Extraction and Real Time PCR Analysis

Tissue samples and cells were treated with TRIzol (Thermo Fisher Scientific, Waltham, MA, USA) and chloroform (Sigma), and total RNA was prepared with an RNA extraction kit (Qiagen; Hilden, Germany). cDNA was synthesized from RNA using a RevertAid First Strand cDNA Synthesis Kit (Thermo Fisher Scientific) with oligo dT. cDNA was amplified with the primer sets shown below and qPCR SYBR Green pre‐mix (Enzynomics; Daejeon, Republic of Korea) on a LightCycler 480 system (Roche).

### Sequences of Real Time PCR Primers

**Table:** 

Species	Name	Forward (5′→3′)	Reverse (5′→3′)
Human	β‐actin	AGAGCTACGAGCTGCCTGAC	AGCACTGTGTTGGCGTACAG
Human	TRAP1	AGCGCACTCATCAGGAAACT	TCAAACTCACGAAGGTGCAG
Human	HIF1α	GAAAGCGCAAGTCCTCAAAG	TGGGTAGGAGATGGAGATGC
Human	ANGPTL4	GGGTCTGGAGGAGGTGCATA	AGTACTGGCCGTTGAGGTTG
Human	VEGF	CTACCTCCACCATGCCAAGT	CTCGATTGGATGGCAGTAGC
Human	ANG1	AGGCTTGGTTTCTCGTCAGA	TCTGCACAGTCTCGAAATGG
Human	ANG2	TGGGATTTGGTAACCCTTC	AAGTTGGAAGGACCACATG
Human	Calpain‐1	ACATGGAGGCCATCACTTTC	GGTCCACGTTGTTCCACTCT
Mouse	β‐actin	TGTCCACCTTCCAGCAGATGT	AGCTCAGTAACAGTCCGCCTAG
Mouse	TRAP1	GAGGAAAGCCAGTTCTGCAC	GCTCTCCTCCTCCTTGTCCT
Mouse	HIF1α	GGGTACAAGAAACCACCCAT	GAGGCTGTGTCGACTGAGAA
Mouse	ANGPTL4	GGAAAAGATGCACCCTTCAA	TGCTGGATCTTGCTGTTTTG
Mouse	VEGF	TTACTGCTGTACCTCCACC	ACAGGACGGCTTGAAGATG
Mouse	ANG1	AGGCTTGGTTTCTCGTCAGA	TCTGCACAGTCTCGAAATGG
Mouse	ANG2	TCCAAGAGCTCGGTTGCTAT	AGTTGGGGAAGGTCAGTGTG
Mouse	Calpain‐1	ACATTTTACGAGGGCACCTG	CTCCCGGTTGTCATAGTCGT

### Calpain Activity Assay

Calpain enzyme activity was measured using a Calpain Activity Assay Kit (Abcam) according to the manufacturer's instructions. In brief, cell and tissue samples were lysed with the extraction buffer provided with the kit. After homogenization and centrifugation of samples, soluble fractions were collected. A mixture of cell lysate, reaction buffer, and calpain substrate (Ac‐LLY‐AFC) was incubated for 1 h at 37 °C. Fluorescence was measured at *λ*_ex_ = 400 nm and *λ*_em_ = 500 nm using a microplate reader (Synergy neo; Biotek; Winooski, VT, USA).

### Ocular Pharmacokinetic Analysis after Topical Drug Administration

The rabbit pharmacokinetic experiment was approved by the IACUC of Knotus (KNOTUS IACUC 22‐KE‐0109) and conducted in Knotus (Republic of Korea). The mouse pharmacokinetic experiment was approved by the IACUC of UNIST. Male New Zealand white rabbits weighing 2.0–2.5 kg provided by the Hallym animal center (Anyang, Republic of Korea), and C57BL/6J mice at P17 were used for the pharmacokinetic study. For topical treatment, 50 and 10 µl SB‐U015 dissolved in Liposic were topically administered once to both eyes of rabbits and mice, respectively. Residual drugs were wiped out after blinking 2–3 times. Animals were sacrificed at 0, 0.5, 1, 2, and 4 h after drug treatment, and the following samples were immediately prepared: retina for mice and rabbits, and cornea, sclera, aqueous humor, and plasma for rabbits. Collected samples were kept at −80 °C until analysis.

Plasma and tissue samples were analyzed according to an optimized bioanalytical method. Ocular tissues (retina, cornea, and sclera) were diluted fivefold with acetonitrile and homogenized with a Retsch MM400 instrument (Haan, Germany). Thereafter, 10 µl aliquots of chlorpropamide were added as an internal standard to 50 µl aliquots of plasma, aqueous humor, and diluted ocular tissues, followed by 200 µl aliquots of acetonitrile for protein precipitation. The mixture was vortexed for 10 min and centrifuged at 16 100 g at 4 °C for 10 min. In total, 5 µl of the supernatant was injected into the LC‐MS/MS system.

The HP 1290 infinity system (Agilent; Santa Clara, CA, USA) was composed of a binary pump, degasser, autosampler, and column oven. A Kinetex C18 column (50 × 2.1 mm, 2.6 µm particle size; Phenomenex; Torrance, CA, USA) was used with a mobile phase consisting of (A) acetonitrile containing 0.1% formic acid and (B) deionized water containing 0.1% formic acid with isocratic mode (A:B = 60:40). The positive ion mode of the Agilent 6430 Triple Quad LC‐MS/MS system (Agilent) linked with ultraperformance liquid chromatography was used. Multiple‐reaction monitoring was used to track the ion transition at m/z 553.2→262.1 for SB‐U015 and m/z 227.0→175.0 for chlorpropamide. MassHunter software (ver 6.0; Agilent) was used to operate LC‐MS/MS and collect data.

Pharmacokinetic analysis was achieved by noncompartmental analysis using Phoenix WinNonlin 8.1 (Certara; Princeton, NJ, USA). The time (T_max_) taken to reach the peak concentration (*C*_max_) was determined directly from the profile of the time‒plasma concentration. The linear‐log trapezoidal rule was applied to calculate the area under the tissue concentration‒time curve from time zero to the last quantification (AUC_last_).

### Statistical Analysis

All data are presented as mean ± standard error of the mean (SEM) from at least two independent experiments. Statistical analyses were performed using Prism 7 (GraphPad Software; La Jolla, CA, USA). The two‐tailed Student's *t*‐test was performed to compare different groups. *P* < 0.05 was considered statistically significant.

## Conflict of interest

B.H.K. is the founder of SmartinBio. Inc. Other authors declare that they have no competing interests.

## Author Contributions

S.Y.K. and N.G.Y. contributed equally to this work. S.Y.K., N.G.Y., D.H.P., and B.H.K. conceived and designed the study. S.Y.K. and N.G.Y. performed most experiments. J.Y.I. generated OIR and STZ mice. Ji Hye Lee contributed to histologic sample preparation. J.K., Y.J.J., and D.H.P. performed OIR mouse experiments. Y.J.C. and Jong‐Hwa Lee performed ocular pharmacokinetics analysis. S.Y.K., N.G.Y., D.H.P., A.U., and B.H.K. analyzed the data. S.Y.K., N.G.Y., D.H.P., and B.H.K. wrote the manuscript with feedback from all authors.

## Supporting information

Supporting Information

## Data Availability

The data that support the findings of this study are available in the supplementary material of this article.
