# Supplementary material for: Targeting the Mitochondrial Chaperone TRAP1 Alleviates Vascular Pathologies in Ischemic Retinopathy
Source: Adv Sci (Weinh). 2023 Nov 20;11(2):2302776. doi: 10.1002/advs.202302776 (PMC10787068; doi:10.1002/advs.202302776)
Supplement: Supplementary file 1 — Supporting Information [file ADVS-11-2302776-s001.pdf]

ADVANCED SCIENCE
Open Access

Supporting Information

for *Adv. Sci.*, DOI 10.1002/advs.202302776

Targeting the Mitochondrial Chaperone TRAP1 Alleviates Vascular Pathologies in Ischemic Retinopathy

So-Yeon Kim, Nam Gu Yoon, Jin Young Im, Ji Hye Lee, Juhee Kim, Yujin Jeon, Young Jae Choi, Jong-Hwa Lee, Akiyoshi Uemura, Dong Ho Park and Byoung Heon Kang**

Supporting information

Targeting the mitochondrial chaperone TRAP1 alleviates vascular pathologies in ischemic retinopathy

Authors: So-Yeon Kim, Nam Gu Yoon, Jin Young Im, Ji Hye Lee, Juhee Kim, Yu Jin Jeon, Young Jae Choi, Jong-Hwa Lee, Akiyoshi Uemura, Dong Ho Park, Byoung Heon Kang**

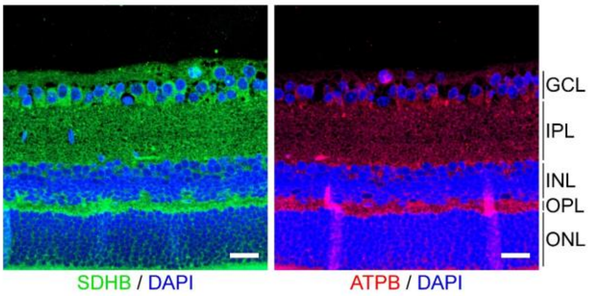

Supplementary Figure 1. Mitochondria staining in mouse retina.

Immunofluorescence staining revealed that SDHB (succinate dehydrogenase subunit B) and ATPB (Complex V beta subunit) were primarily detected in both inner plexiform layer (IPL) and outer plexiform layer (OPL), but were largely absent from the inner nuclear layer (INL) and outer nuclear layer (ONL). Scale bar, 20 μm.

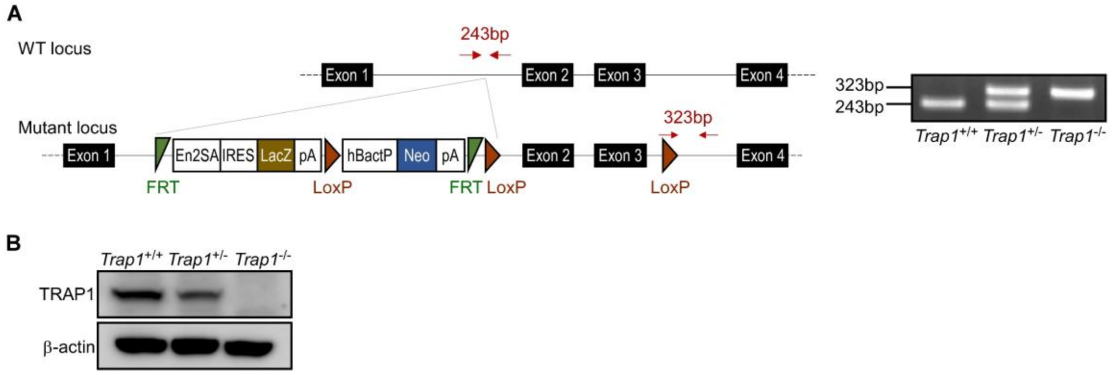

Supplementary Figure 2. Generation of *Trap1* knockout (*Trap1*$^{-/-}$) mice.

A) Gene trap *Trap1*-KO locus. The gene trap cassette [1] was inserted in the intron between exons 1 and 2 of *Trap1* in chromosome 16 of a C57BL/6 mouse. Red arrows indicate the locations of the primers for detection of the wild-type and KO alleles (left), which generated PCR products of 243 and 323 bp, respectively (right). FRT, Flp recognition target; En2SA, engrailed-2 splicing acceptor; IRES, internal ribosome entry site; LacZ, lacZ β-galactosidase; pA, polyadenylation signal; LoxP, locus of X-over P1; hBactP, human β-actin promoter; Neo, neomycin. **B**) TRAP1 expression in *Trap1*$^{-/-}$ mice. Western blot analysis was performed to measure retinal TRAP1 levels in *Trap1*$^{+/+}$, *Trap1*$^{+/-}$, and *Trap1*$^{-/-}$ mice.

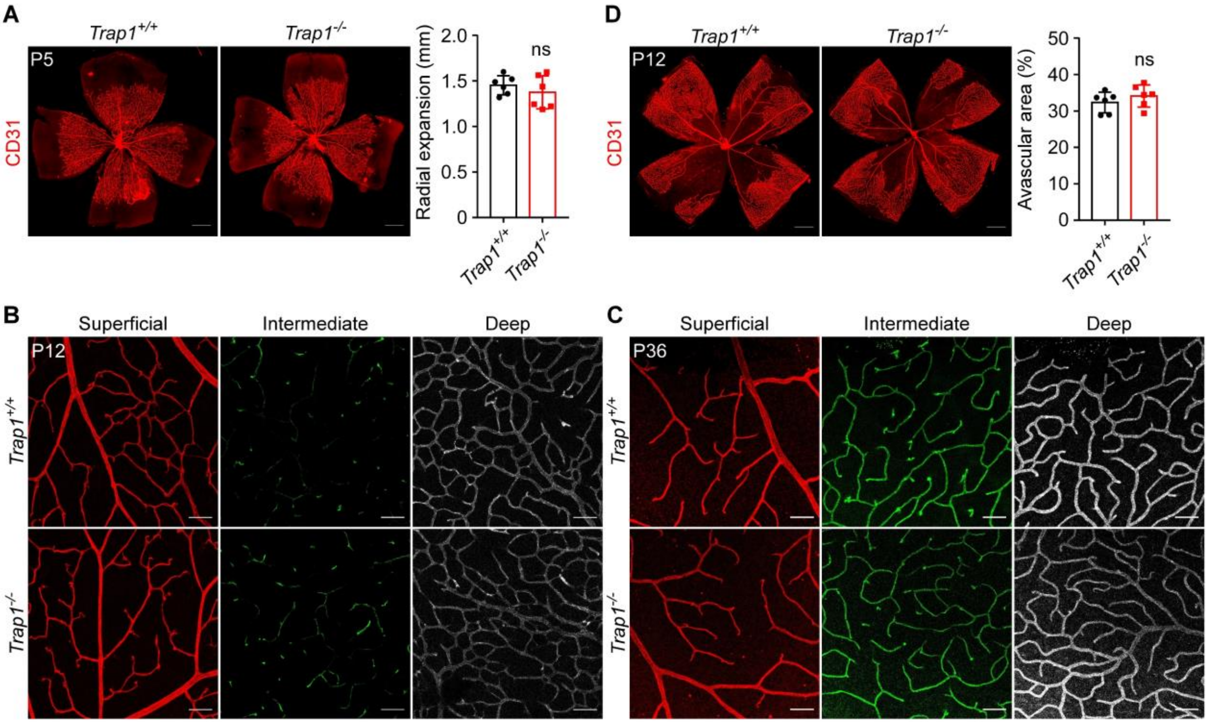

Supplementary Figure 3. *Trap1* KO does not affect physiological angiogenesis or hyperoxia-induced vessel regression in mouse retinas.

A) Whole-mount retinal staining of CD31 at P5. **Left**. Mouse retinas were collected at P5 and stained with anti-CD31 antibody to analyze physiological vessel development. Retinal images indicate that radial expansion of blood vessels did not differ significantly between *Trap1*$^{+/+}$ and *Trap1*$^{-/-}$ mice. Scale bar, 500 μm. **Right**. Quantification of radial expansion in retinas of P5 *Trap1*$^{+/+}$ and *Trap1*$^{-/-}$ mice (n = 6 mice/group). **B,C)** Whole-mount retinal staining with anti-CD31 antibody at P12 and P36. Mouse retinas were collected at P12 and P36 and stained using anti-CD31 antibody to analyze physiological vessel development. Vessel development was comparable between *Trap1*$^{+/+}$ and *Trap1*$^{-/-}$ mice in the superficial, intermediate, and deep vascular layers of the retina. Scale bar, 50 μm. **D**) Effect of hyperoxia on retinal vasculature. **Left**. Retinas collected from *Trap1*$^{+/+}$ and *Trap1*$^{-/-}$ OIR mice at P12, directly following 5-day hyperoxia exposure, were prepared as whole-mounts and stained with anti-CD31 antibody.

Retinal avascular areas were comparable between *Trap1*$^{+/+}$ and *Trap1*$^{-/-}$ mice directly following hyperoxia. **Right**. Quantification of retinal avascular areas at P12 (n = 6 mice/group). Data information: Data are expressed as mean ± SEM. Student *t*-test, ns, not significant.

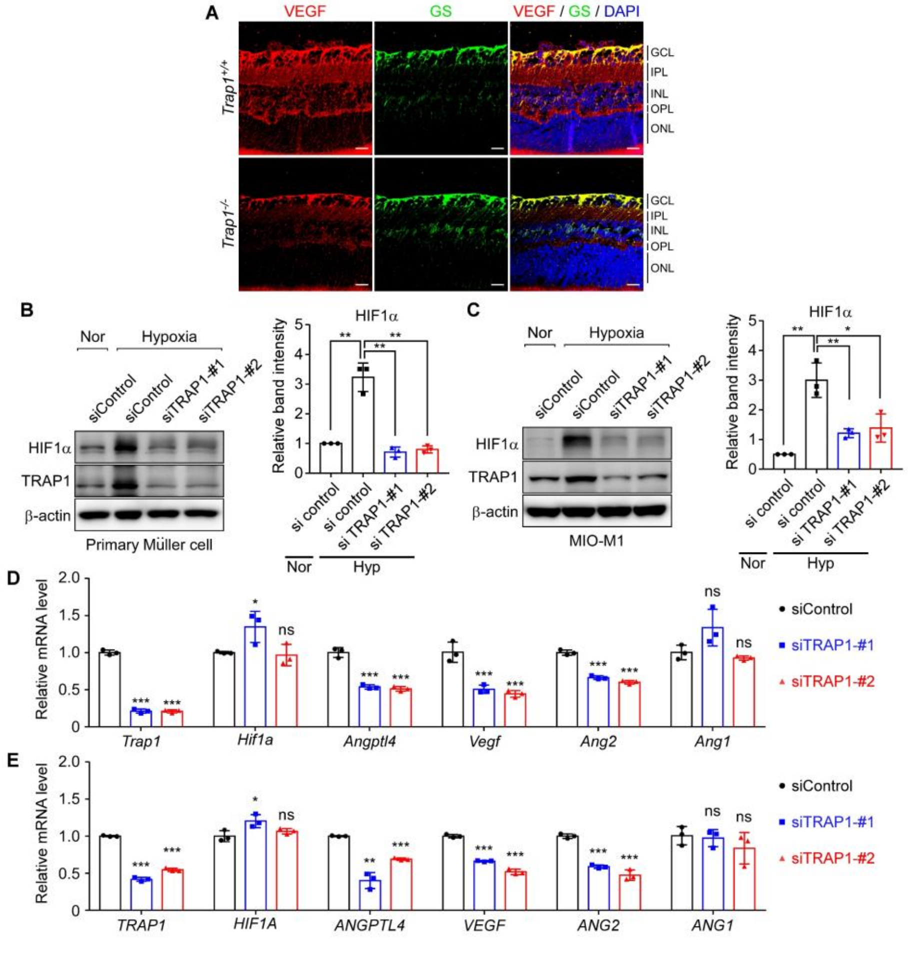

Supplementary Figure 4. TRAP1 regulates HIF1α stabilization and downstream angiogenic factor expression.

A) Immunohistochemical staining of VEGF and glutamine synthetase (GS) in the retinas of

OIR mice at P12. Mouse retinas were collected, fixed, and then stained with antibodies against VEGF (red) and GS (green, Müller cell marker). Scale bar, 20 μm. **B**) TRAP1 depletion in primary Müller cells under hypoxia. **Left**. Primary Müller cells prepared from mouse retina were treated with TRAP1-targeting siRNAs for 48 hr, exposed to hypoxia for 6 hr, and analyzed by western blotting. **Right**. The band intensities of HIF1α were normalized to those of β-actin and comparatively analyzed. **C**) TRAP1 depletion in the human Müller cell line MIO-M1 under hypoxia. Three independent experiments were performed as in (**B**) with MIO-M1 cells. **D**) Quantitation of angiogenic factors in mouse primary Müller cells. Primary Müller cells were incubated with TRAP1-targeting siRNAs for 48 hr, exposed to hypoxia for 6 hr, and analyzed by qPCR. **E**) Quantitation of angiogenic factors in MIO-M1 cells. Three independent experiments were performed as in (**D**) with MIO-M1 cells. Data information: Nor, normoxia; Hyp, hypoxia; Data are expressed as mean ± SEM from three independent experiments. Student's *t*-test, ****P* < 0.001; ***P* < 0.01; **P* < 0.05; ns, not significant.

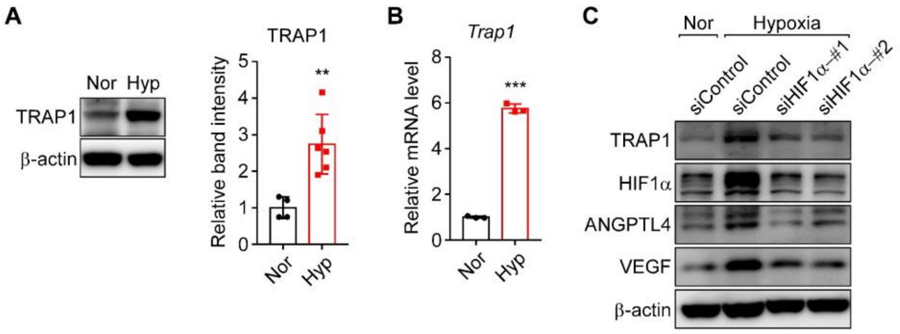

Supplementary Figure 5. HIF1α regulates TRAP1 expression.

A) TRAP1 protein expression in mouse primary Müller cells. **Left.** Primary Müller cells obtained from mice were exposed to normoxia (Nor) or hypoxia (Hyp) for 6 hr, and analyzed by western blotting. **Right**. The band intensities of TRAP1 were normalized to those of β-actin and compared. The data are mean ± SEM from at least four independent experiments. **B**) *Trap1* mRNA expression in mouse primary Müller cells. Cells were treated as in (**A**), and *Trap1* mRNA expression was comparatively analyzed by qPCR. The data are mean ± SEM from three independent experiments. **C**) HIF1α regulates TRAP1 expression. Mouse primary Müller cells were treated with control and HIF1α-targeting siRNAs for 48 hr, exposed to normoxia or hypoxia for 6 hr, and analyzed by western blotting. Data information: Data are expressed as mean ± SEM. Student's *t*-test, ***$P < 0.001$; **$P < 0.01$.

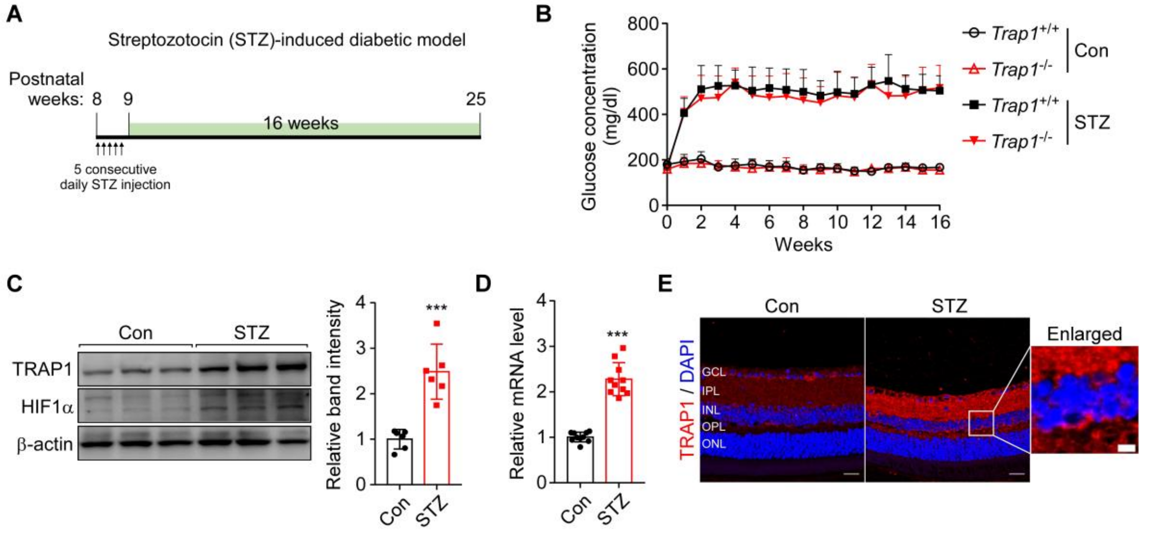

Supplementary Figure 6. Expression of TRAP1 in STZ mouse retinas.

A) Schematic of the experimental procedures for the production of streptozotocin (STZ) treated mice. STZ mice were generated as described in the Experimental Section. For the STZ-induced diabetic model, 8-week-old male mice were injected intraperitoneally with 75 mg/kg STZ for 5 consecutive days. At 16 weeks after STZ injection, mouse eyes were collected for analyses. **B)** Measurement of blood glucose concentration. Blood glucose concentration was measured in *Trap1*$^{+/+}$ and *Trap1*$^{-/-}$ mice that were either treated with STZ (STZ) or untreated (Con). (n = 10 mice for Con group, n = 20 mice for STZ group) **C)** TRAP1 expression in STZ mouse retinas. **Left**. Retinas collected from STZ mice and age-matched control mice (Con, vehicle treatment) were analyzed by western blotting. **Right**. Quantification of the TRAP1 protein level. The band intensities of TRAP1 were normalized to those of β-actin in STZ mouse retinas and compared. (n = 6mice/group) **D**) Quantification of TRAP1 mRNA. OIR mouse retinas were comparatively analyzed by quantitative real time PCR (qPCR) (n = 10; duplicate experiment of 5 mice/group). **E**) Immunohistochemical

staining of TRAP1 in mouse retinas. Collected mouse retinas were fixed and stained with an anti-TRAP1 antibody (red). Scale bar, 20 μm and 5 μm (enlarged image). (n = 6 mice/group)

Data information: Data are expressed as mean ± SEM. Student *t*-test, ***$P < 0.001$.

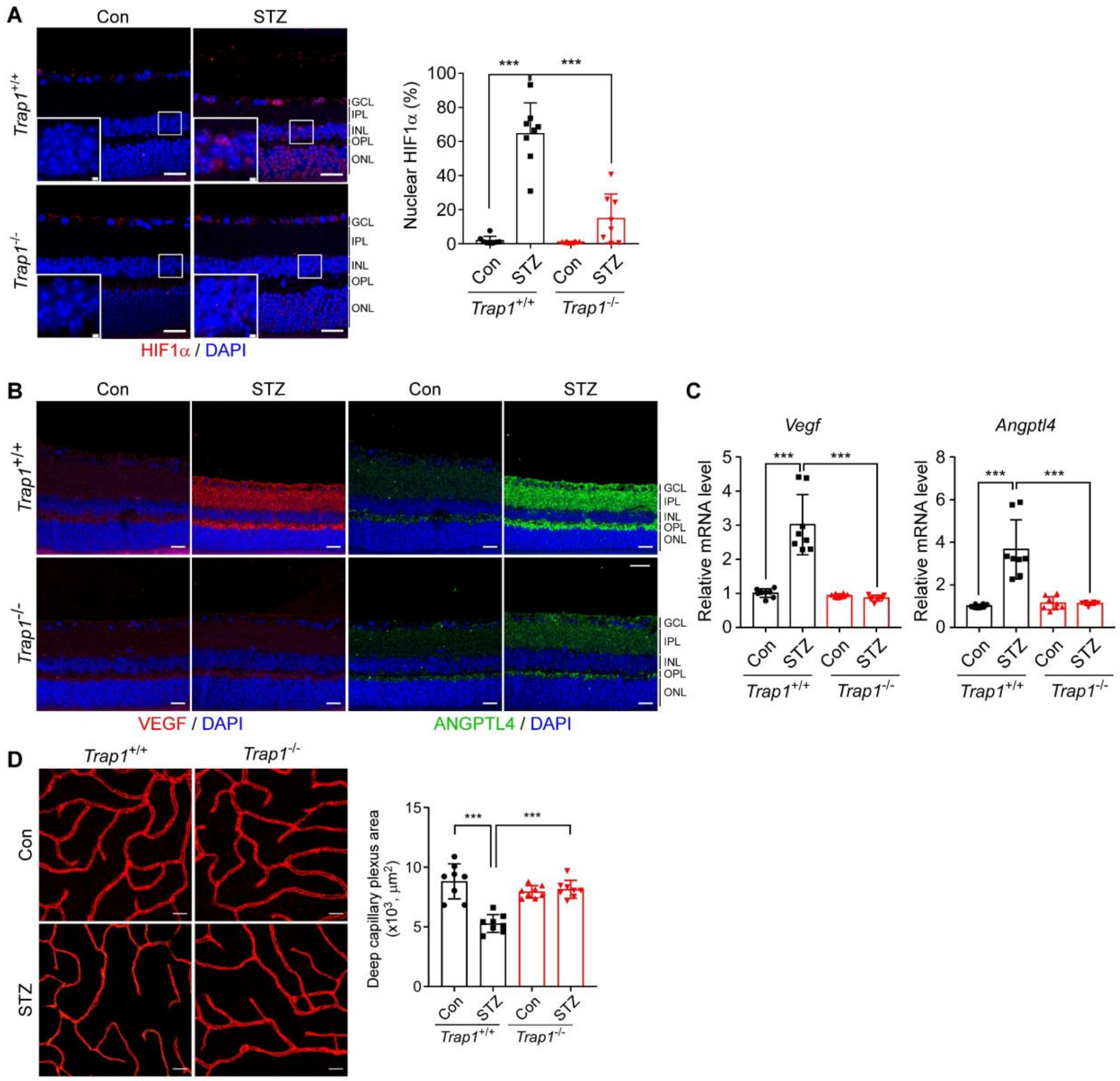

Supplementary Figure 7. *Trap1* KO reduces the expression of HIF1α and angiogenic factors while increasing vessel density in the STZ mice.

A) HIF1α expression in STZ mouse retinas. **Left**. Retinal sections collected from $\textit{Trap1}^{+/+}$ and $\textit{Trap1}^{-/-}$ STZ mice were stained with an anti-HIF1α antibody (red) and DAPI (blue), and imaged by confocal microscopy. Scale bars, 20 μm and 2 μm (inset). **Right**. Quantification of confocal microscopic images. The ratio (%) of HIF1α and DAPI double-positive areas to DAPI-positive areas was calculated (n = 8 mice/group). **B**) VEGF (red) and ANGPTL4 (green) expression in STZ mouse retinas. Retinal sections collected from $\textit{Trap1}^{+/+}$ and $\textit{Trap1}^{-/-}$ mice treated with vehicle (Con) or STZ were analyzed by immunofluorescence staining and confocal microscopy (n = 3 mice/group). Scale bar, 20 μm. **C**) Quantitation of mRNA levels in STZ mouse retinas. Retinas from $\textit{Trap1}^{+/+}$ and $\textit{Trap1}^{-/-}$ mice treated with vehicle (Con) and STZ were comparatively analyzed by qPCR (n = 8; duplicate experiment of 4 mice/group). **D**) Vessel density of the deep capillary plexus in STZ mouse retinas. **Left**. Whole-mount staining of retinas from $\textit{Trap1}^{+/+}$ and $\textit{Trap1}^{-/-}$ STZ mice using an anti-CD31 antibody. Scale bar, 20 μm. **Right**. Quantification of CD31 positive areas (n = 5 mice/group). Data information: Data are expressed as mean ± SEM. Student *t*-test, $***P < 0.001$.

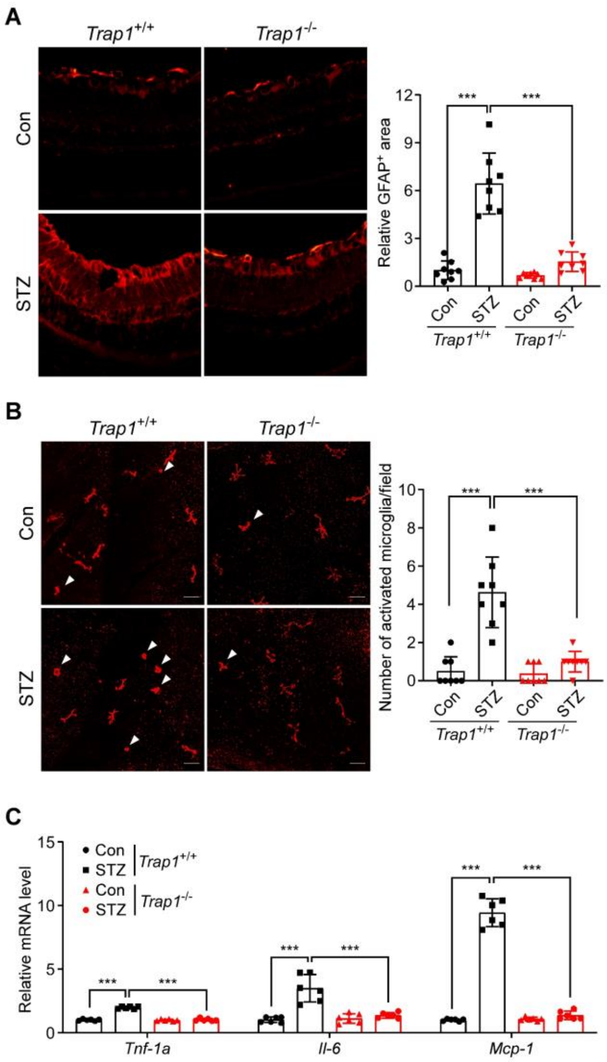

Supplementary Figure 8. Activated Müller glia and microglia were reduced in TRAP1$^{-/-}$ mice

A) Activation of Müller glia in STZ mouse retinas. **Left**. Retinal sections collected from *Trap1*$^{+/+}$ and *Trap1*$^{-/-}$ STZ mice were stained with an anti-GFAP antibody, and analyzed by fluorescence microscopy. Scale bars, 20 µm. **Right**. Quantification of microscopic images. The GFAP positive area was calculated (n = 8; duplicate experiments of 4 mice/group). **B)**

Activation of microglia in STZ mouse retinas. **Left**. Whole-mount retinas collected from *Trap1*$^{+/+}$ and *Trap1*$^{-/-}$ STZ mice were stained with an anti-F4/80 antibody, and analyzed by confocal microscopy. Scale bars, 50 μm. **Right**. Quantification of microscopic images. The activated amoeboid-shape microglia were calculated (n = 8; duplicate experiments of 4 mice/group). **C)** Quantitation of inflammatory cytokine mRNA levels in STZ mouse retinas. Retinas from *Trap1*$^{+/+}$ and *Trap1*$^{-/-}$ mice treated with vehicle (Con) or STZ were comparatively analyzed by qPCR (n = 6; duplicate experiments of 3 mice/group). Data information: Data are expressed as mean ± SEM. Student *t*-test, ***$P < 0.001$.

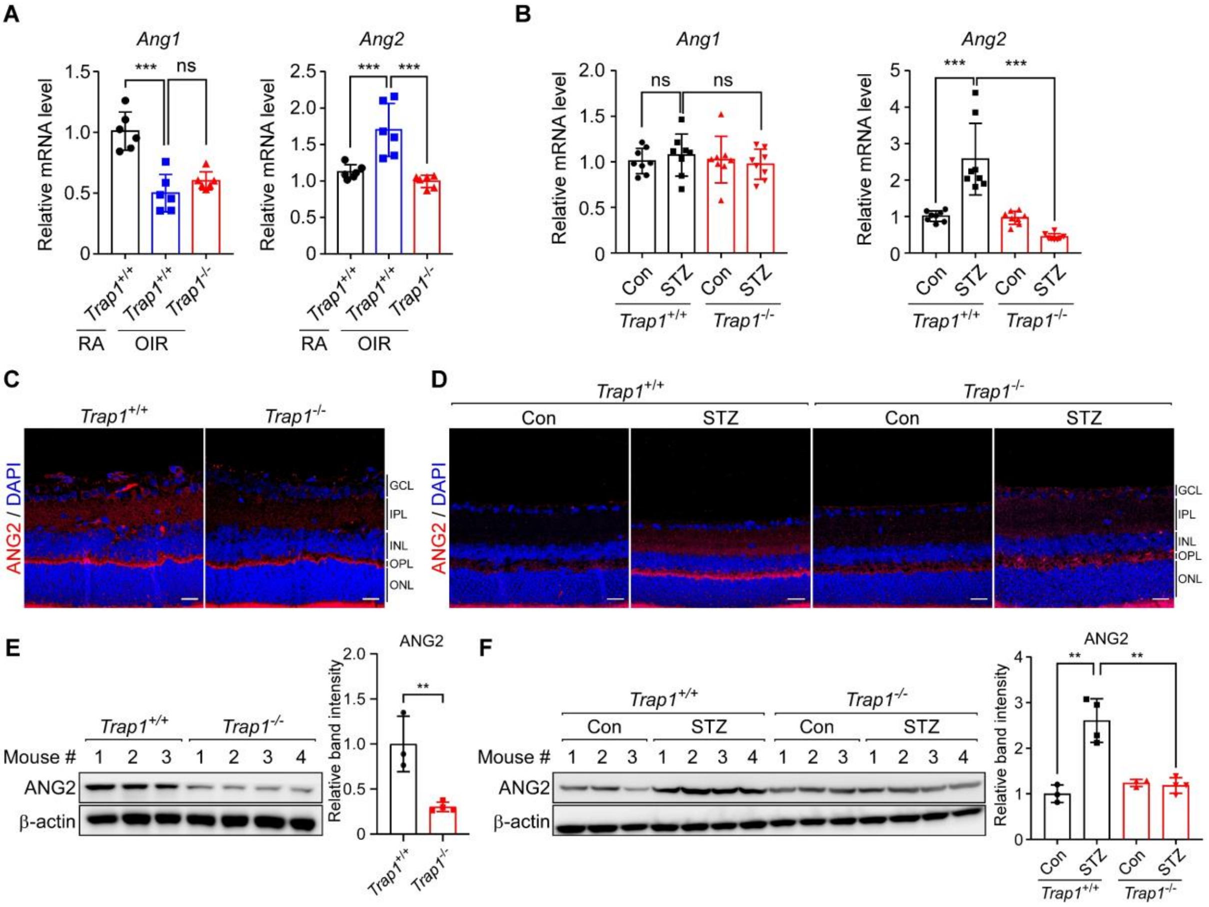

Supplementary Figure 9. ANG2 expression is down-regulated in *Trap1*$^{-/-}$ mice.

A,B) *Ang1* and *Ang2* mRNA levels in OIR (**A**) and STZ (**B**) mouse retinas. Retinal samples obtained from *Trap1*$^{+/+}$ and *Trap1*$^{-/-}$ OIR mice (n = 6; duplicate experiments of 3 mice/group) and STZ (n = 8; duplicate experiments of 4 mice/group) mice were analyzed by qPCR to measure *Ang1* and *Ang2* mRNA levels. *Ang2* mRNA expression was dramatically reduced in both OIR and STZ mouse retinas with the *Trap1*-KO genotype (*Trap1*$^{-/-}$); however, *Ang1* mRNA expression was not affected by *Trap1*$^{-/-}$. **C,D**) ANG2 expression in OIR and STZ mouse retinas. Retinal sections obtained from *Trap1*$^{+/+}$ and *Trap1*$^{-/-}$ OIR (**C**) and STZ (**D**) mice were analyzed by immunofluorescence staining. Representative images from three independent experiments are shown. Scale bar, 20 µm. Elevated ANG2 expression in the IPL and OPL of OIR and STZ mice was dramatically reduced by *Trap1*-KO. **E**) ANG2 protein

level in OIR mouse retinas. **Left.** Retinas collected from *Trap1*$^{+/+}$ (n = 3 mice) and *Trap1*$^{-/-}$ (n = 4 mice) OIR mice were analyzed by western blotting. **Right.** Protein band intensities of ANG2 were normalized to those of β-actin and comparatively analyzed. **F**) ANG2 protein level in STZ mouse retinas. **Left.** Retinal samples collected from STZ (n = 4 mice) and age-matched control (n = 3 mice) mice with the *Trap1*$^{+/+}$ or *Trap1*$^{-/-}$ genotype were analyzed by western blotting. **Right.** Protein band intensities were analyzed as in (**E**). Data information: Data are expressed as mean ± SEM. Student's *t*-test, $***P < 0.001$; $**P < 0.01$; ns, not significant.

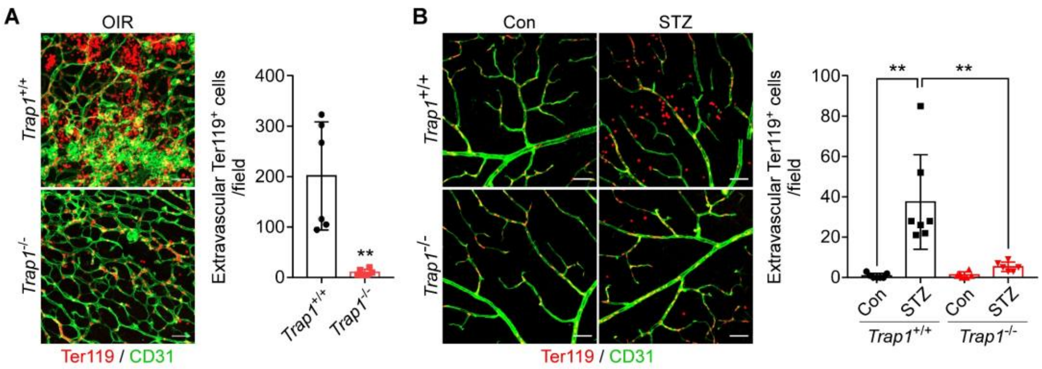

Supplementary Figure 10. Decreased extravasations of red blood cells in *Trap1*$^{-/-}$ mice.

A, B) Red blood cell staining of OIR and STZ mouse retinas. **Left.** Red blood cells and blood vessels were stained with anti-Ter119 (red) and anti-CD31 (green) antibodies in whole-mount retinas collected from OIR (**A**) (n = 6; 3 mice/group, 2 fields/mouse) and STZ mice (**B**) (n = 6–7; 3 mice/group, 2–3 fields/mouse). Scale bar, 50 μm. **Right**. Quantification of extravascular Ter119 positive cells. Extravasation of red blood cells was reduced in *Trap1*$^{-/-}$ mice. Data information: Data are expressed as mean ± SEM. Student's *t*-test, $**P < 0.01$

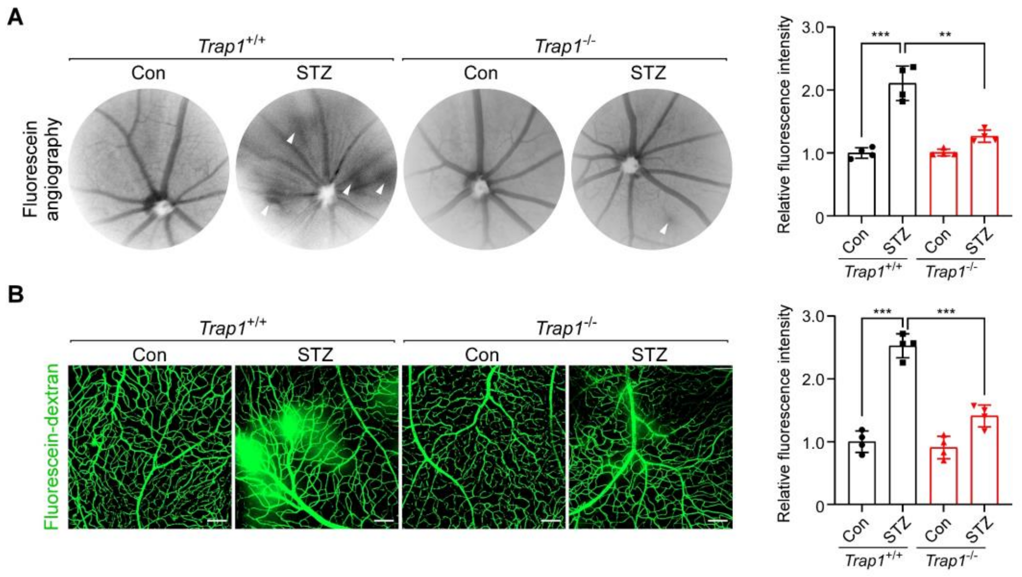

Supplementary Figure 11. Retinal vascular leakages were reduced in TRAP1$^{-/-}$ mice

A) Fluorescein angiography of STZ mouse retinas. **Left.** Fluorescein was intraperitoneally injected into live STZ mice, and images were captured using the iVivo Funduscope. Vascular leakages are indicated with white arrows. **Right**. Quantification of fluorescence intensity (n = 4; 4 mice/group). **B)** Vascular leakages in STZ mouse retinas. **Left.** After intravenous injection of fluorescein-dextran, the retinas were collected, whole-mounted, and analyzed using confocal microscopy. Scale bar, 100 μm. **Right**. Quantification of fluorescence intensity

(n = 4; 4 mice/group). Data information: Data are expressed as mean ± SEM. Student's *t*-test, ***$P < 0.001$, **$P < 0.01$

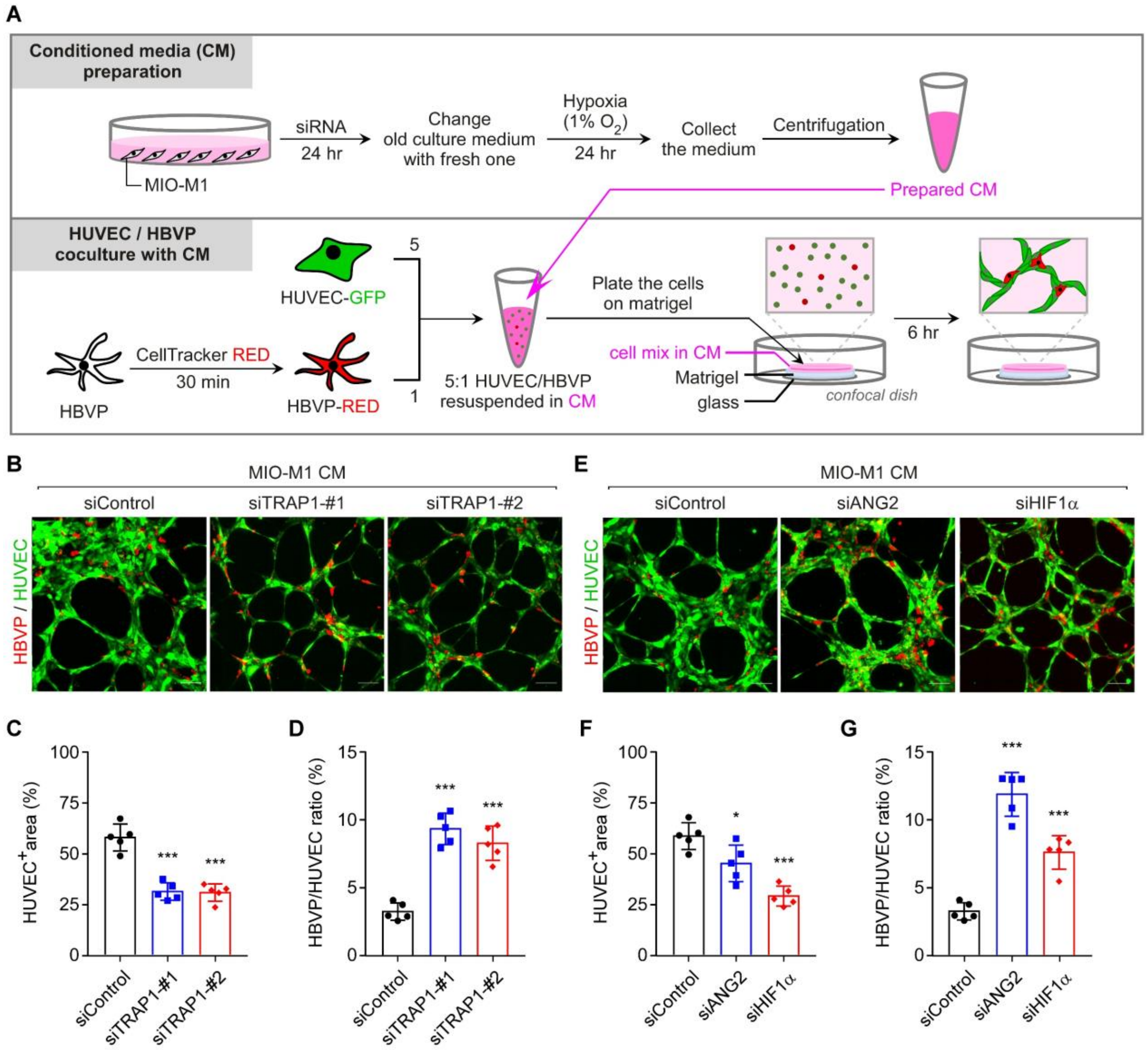

Supplementary Figure 12. TRAP1 inhibition normalizes pericyte-endothelial cell interactions.

A) Experimental scheme for tube formation assay. MIO-M1 cells were incubated with control or TRAP1-targeting siRNAs for 24 hr. After exchanging the medium with fresh medium, cells were incubated for 24 hr under hypoxia to obtain CM. The tube formation assay was

performed by coculturing HUVECs and HBVPs. GFP-expressing HUVECs and CellTracker Red CMTPX-labeled HBVPs at a ratio of 5:1 were resuspended in the collected CM and plated on a Matrigel-coated confocal plate, incubated for 6 hr, and analyzed by confocal microscopy. **B**) Tube formation upon treatment with CM collected from control and TRAP1-targeting siRNA-treated MIO-M1 cells. Scale bar, 100 μm. **C**) Quantification of the GFP-positive (HUVECs) area per field. Confocal microscopic images were analyzed to quantify the percentage area of HUVECs (green fluorescence) per field (n = 5). **D**) Quantification of the ratio of red (HBVPs) to green (HUVECs) fluorescence areas (n = 5). **E)** Tube formation upon treatment with CM collected from HIF1α- and ANG-2-targeting siRNA-treated MIO-M1 cells. Scale bar, 100 μm. **F**) Quantification of the GFP-positive area per field (n = 5). **G**) Quantification of the ratio of red (HBVPs) to green (HUVECs) fluorescence areas (n = 5). Data information: Data are expressed as mean ± SEM. Student *t*-test, ***$P < 0.001$; *$P < 0.05$

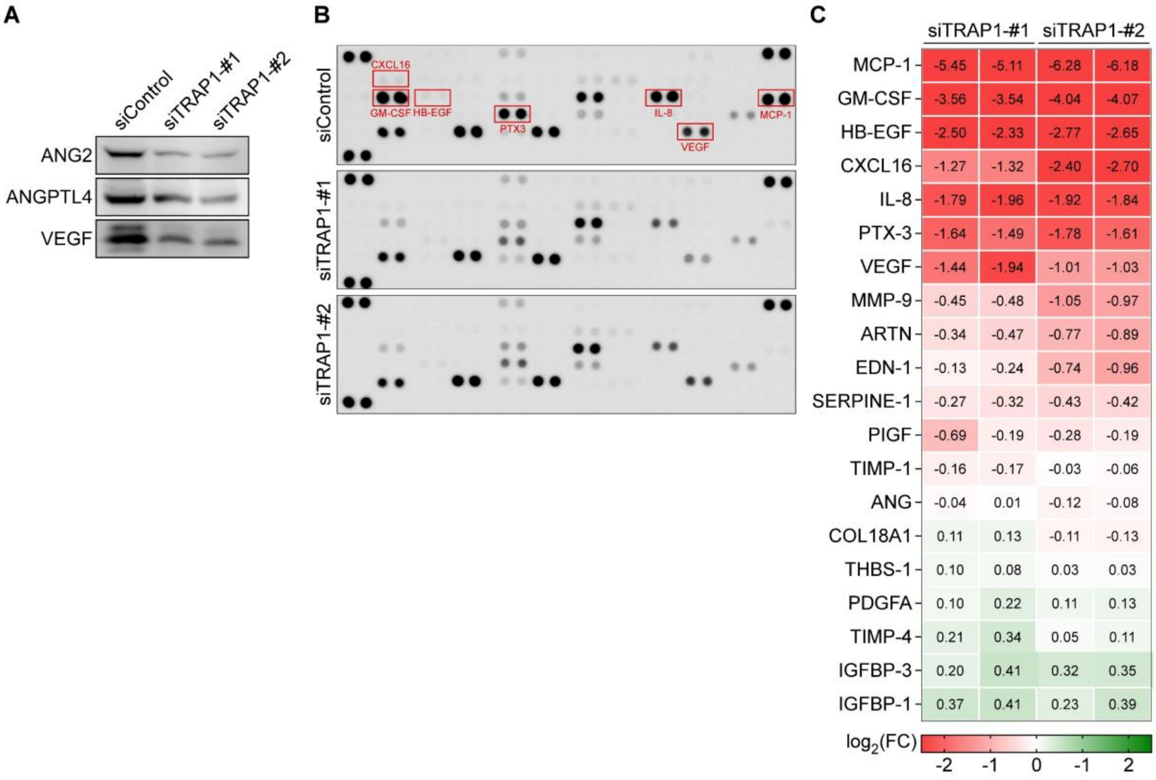

Supplementary Figure 13. Angiogenic mediator expression in conditioned media.

A) Western blot analysis of the conditioned media (CM). MIO-M1 cells were incubated with either control (siControl) or TRAP1 siRNAs (siTRAP1) for 24 hr. Subsequently, the medium was replaced with fresh medium, and the cells were subjected to an additional 24 hr incubation under hypoxia. The medium, now referred to as CM, was then analyzed by western blotting. Elevated expression of VEGF, ANGPTL4, and ANG2 under hypoxia was reduced after TRAP1 inhibition.

B) Human angiogenesis antibody array analysis. The CM obtained in (**A**) was analyzed using the Proteome Profiler Human Angiogenesis Array Kit (R&D Systems) to assess changes in angiogenesis-related factors, following the manufacturer's instructions. The chemiluminescence signals were then captured using a luminescent image analyzer.

C) Quantification of spot intensity. Each protein is represented by duplicated spots in (**B**). The

spot intensities were normalized to that of positive control on the same membrane. The normalized spot intensities from siTRAP1 samples were then compared with those of siControl samples.

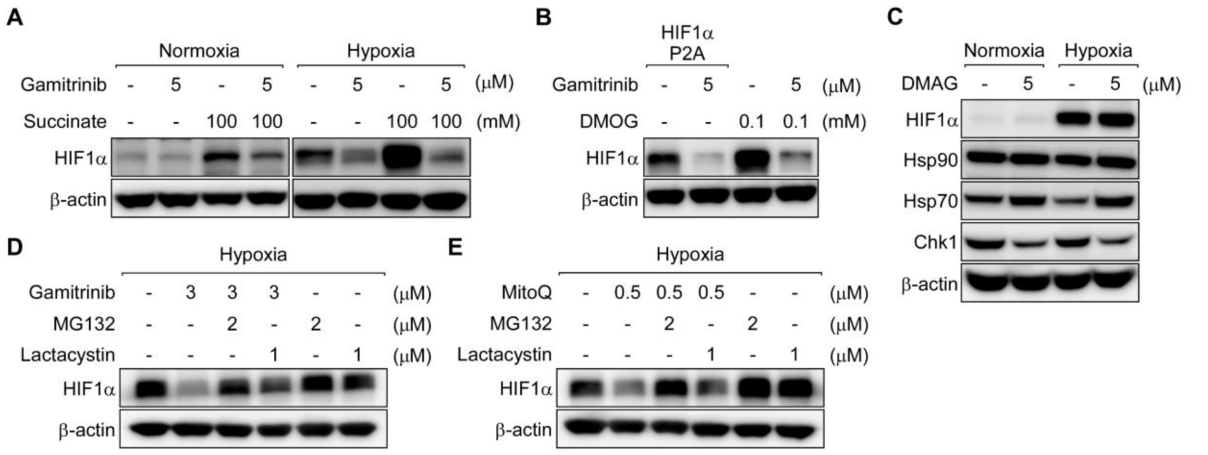

Supplementary Figure 14. TRAP1 regulates HIF1α stability independently of PHD, Hsp90, and the proteasome.

A) Effect of succinate on the TRAP1-HIF1α pathway. MIO-M1 cells were incubated with succinate and the TRAP1 inhibitor Gamitrinib [2] as indicated for 6 hr under normoxia or hypoxia, and analyzed by western blotting. Exposure to a large excess concentration of succinate did not affect gamitrinib-induced HIF1α degradation. **B**) Inhibition of HIF1α hydroxylation. MIO-M1 cells were transfected with HIF1α P2A (both proline hydroxylation sites at positions 402 and 564 were mutated to alanine) for 24 hr and then incubated with gamitrinib for 6 hr. Alternatively, MIO-M1 cells were incubated with DMOG (a PHD

inhibitor) and gamitrinib as indicated for 6 hr, harvested, and analyzed by western blotting. Proline mutation and DMOG treatment did not affect gamitrinib-induced HIF1α degradation, indicating the mechanism is PHD-independent. **C**) Cytoplasmic Hsp90 inhibition. MIO-M1 cells were incubated with DMAG (an Hsp90 inhibitor) as indicated for 6 hr under normoxia or hypoxia and analyzed by western blotting. DMAG treatment downregulated the Hps90 client protein checkpoint kinase 1 (Chk1) and upregulated the stress response protein Hsp70, indicating it inhibited cytoplasmic Hsp90, but did not affect HIF1α stability. The data indicate that cytoplasmic Hsp90 does not affect the stability of HIF1α in MIO-M1 cells. **D,E**) Proteasomal inhibitors. MIO-M1 cells were incubated with gamitrinib or the allosteric TRAP1 inhibitor MitoQ [3] in the presence or absence of MG132 or lactacystin under hypoxia and analyzed by western blotting. The HIF1α level reduced by TRAP1 inhibitors was not effectively restored by lactacystin, but was completely restored by MG132.

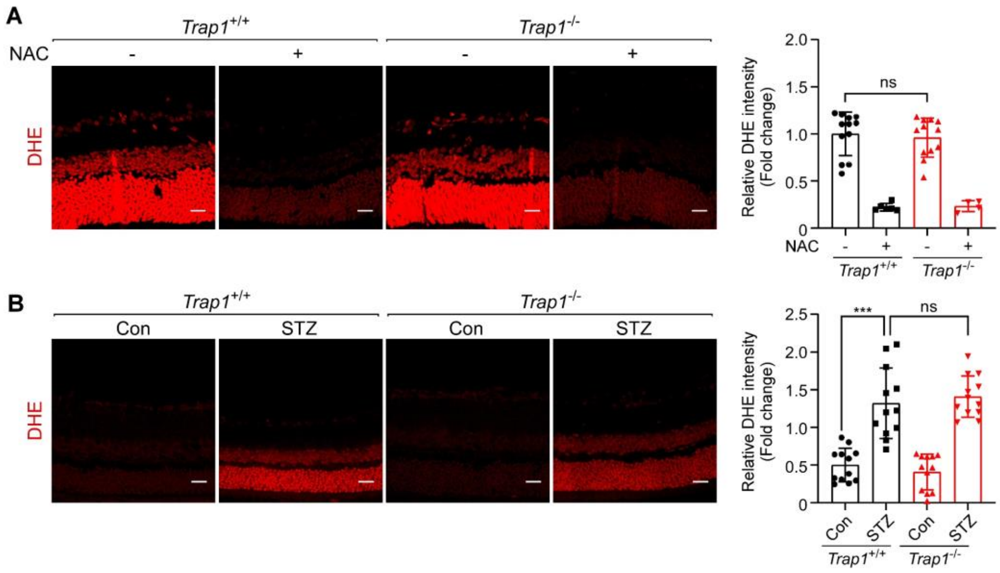

Supplementary Figure 15. Inhibition of TRAP1 does not affect retinal ROS levels

A,B) Dihydroethidium (DHE) staining of OIR and STZ mouse retinas. **Left.** Retinal sections collected from OIR (**A**) (n = 12; 6 mice/group, 2 fields/mouse) and STZ mice (**B**) (n = 12; 6 mice/group, 2 fields/mouse) were stained with DHE and examined using fluorescence microscopy. In (**A**), an antioxidant, N-acetyl cysteine (NAC, 10 mM), was used to scavenge ROS, demonstrating ROS-specific DHE staining (n = 4; 4 mice/group). Scale bar, 20 µm. **Right**. Quantification of DHE fluorescence intensity. The fluorescence intensities were calculated and compared, showing no significant changes in ROS staining between *Trap1*$^{+/+}$ and *Trap1*$^{-/-}$ mice. Data information: Data are expressed as mean ± SEM. Student *t*-test, ***$P < 0.001$; ns, not significant.

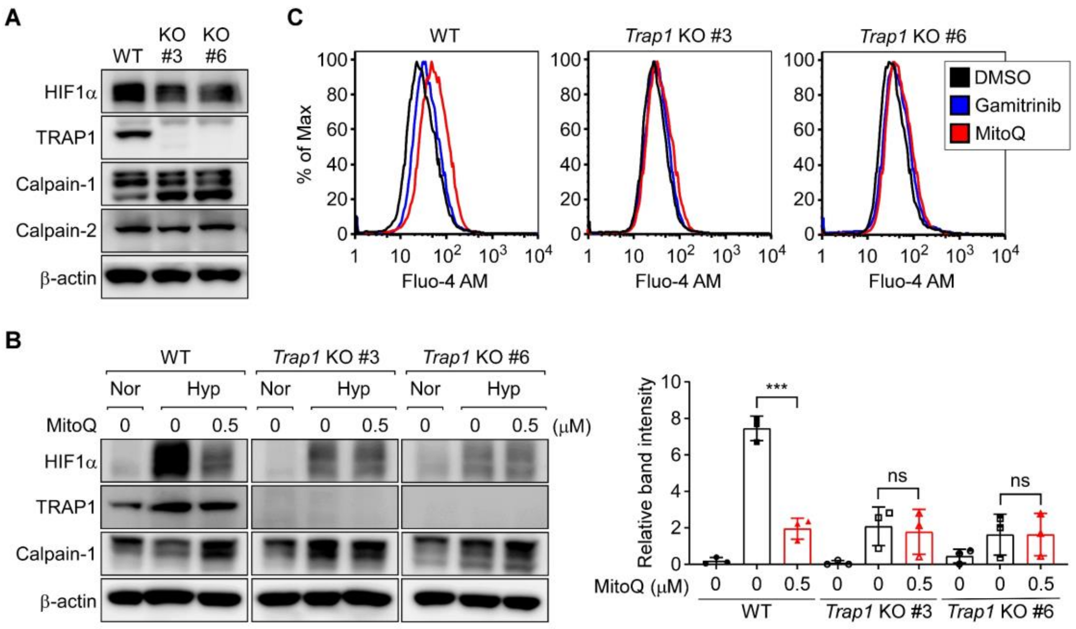

Supplementary Figure 16. Calpain-1 autolysis and HIF1α degradation in *Trap1* knockout cell lines.

A) HIF1α degradation and calpain-1 autolysis in *Trap1* knockout (KO) MIO-M1 cells. *Trap1* KO MIO-M1 cells were generated as described in the Experimental Section and incubated in hypoxia for 6 hr, followed by analysis using western blotting. Under hypoxia, HIF1α degradation and autoproteolysis of calpain-1 were found in *Trap1* KO MIO-M1 cells, when compared with wild type (WT) cells.

B) MitoQ does not trigger HIF1α degradation in *Trap1* KO MIO-M1 cells. **Left**. *Trap1* KO cell lines were established using MIO-M1 cells. WT and KO cells were incubated with MitoQ for 6 hr under hypoxia and analyzed by western blotting. *Trap1* KO reduced the expression of HIF1α in MIO-M1 cells, which was not further affected by MitoQ treatment. **Right.** Quantification of the HIF1α protein level. HIF1α western blot density was analyzed and compared. Data are expressed as mean ± SEM from four independent experiments.

C) Cytoplasmic calcium concentration. WT and *Trap1*-KO MIO-M1 cells were labeled with

Fluo-4 AM, incubated with 3 μM gamitrinib or 1 μM MitoQ for 6 hr under hypoxia, collected, and analyzed by flow cytometry. Data information: Data are expressed as mean ± SEM. Student *t*-test, ****P* < 0.001; ns, not significant.

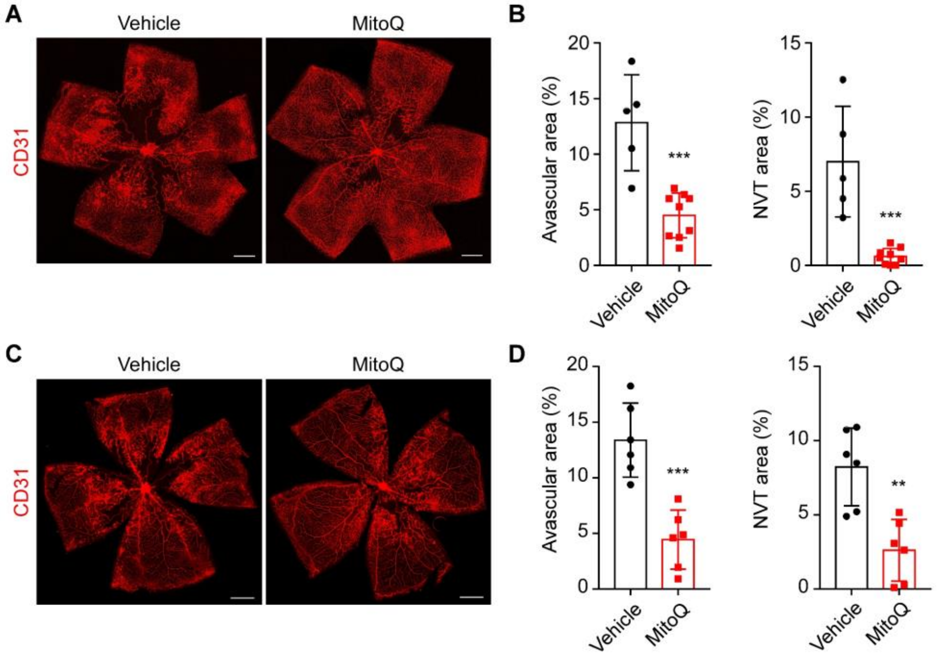

Supplementary Figure 17. Intravitreal injection and topical application of MitoQ reduces avascular and neovascular tuft areas in OIR mice

A,B) Intravitreal injection of MitoQ in OIR mice. In total, 1 μl MitoQ (0.1 mg/ml) was intravitreally injected into OIR mice at P12. Mouse retinas were harvested at P17, flat mounted, and immunohistochemically analyzed using an anti-CD31 antibody. Intravitreal injection of MitoQ significantly reduced avascular and neovascular tuft (NVT) areas. Scale

bar, 500 μm. **B**) Quantification of avascular and neovascular tuft areas in OIR mice intravitreally injected with MitoQ (n = 5 mice/group). **C**) Topical application of MitoQ in OIR mice. In total, 10 μl MitoQ (1 mM) was topically administered three times daily from P12 to P17. Harvested retinal samples were whole-mounted and analyzed as in (**A**). MitoQ administration reduced the avascular and NVT areas in OIR mice as much as *Trap1* KO. Scale bar, 500 μm. **D**) Quantification of avascular and neovascular tuft areas in OIR mice topically treated with MitoQ (n = 6 mice/group). Data information: The data are mean ± SEM. Student's *t*-test, ***$P < 0.001$; **$P < 0.01$.

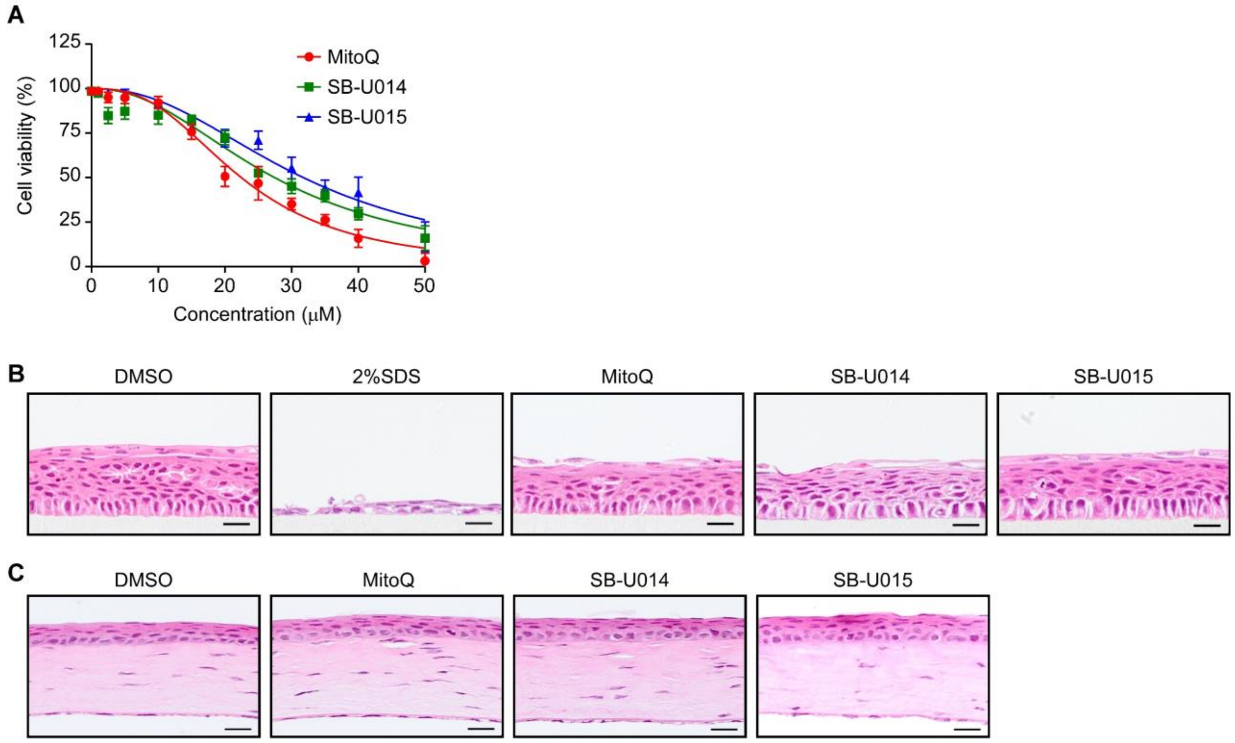

Supplementary Figure 18. Toxicity of TRAP1 inhibitors

A) Normal cell toxicity of TRAP1 inhibitors. Primary Müller cells isolated from mouse retina were treated with MitoQ, SB-U014, or SB-U015 for 24 hr under hypoxia as indicated, and

their cytotoxic activities were analyzed in four independent experiments by trypan blue staining. The calculated IC_{50} values are presented in the normal cell cytotoxicity column in **Table 1**. **B**) Topical toxicity of TRAP1 inhibitors. A 3D culture of human corneal epithelium (MCTT HCE) was incubated with 2 mM TRAP1 inhibitors and analyzed by H&E staining. The culture thickness (n = 5) was measured and analyzed (**Table S1**). Scale bar, 25 μm. **C**) Corneal toxicity. After topical application of 2 mM MitoQ, SB-U014, or SB-U015 once daily for 6 days, the mouse cornea was harvested and analyzed by H&E staining. Treatment with TRAP1 inhibitors did not cause any noticeable abnormality. Scale bar, 25 μm.

Supplementary Table 1. Percentage viability and thickness of the 3D culture of MCTT HCE [4]

Drug	Percentage viability (%)	Thickness (μm)
DMSO	100.00 ± 2.00	58.88 ± 2.21
MitoQ	63.47 ± 0.23	53.43 ± 2.79
SB-U014	27.47 ± 0.56	44.25 ± 3.13
SB-U015	66.90 ± 0.48	55.49 ± 1.29
2% SDS	16.04 ± 1.10	11.03 ± 1.65

A 3D culture of human corneal epithelium (MCTT HCE) was incubated with 2 mM TRAP1 inhibitors, and the percentage viability was analyzed by the WST-1 assay (n = 4). Absorbance was measured with a microplate reader. Thickness was calculated from H&E staining images (n = 5) as in **Figure S18B, Supporting information**. Data Information: The data are expressed as mean ± SEM.

References

[1] W. C. Skarnes, B. Rosen, A. P. West, M. Koutsourakis, W. Bushell, V. Iyer, A. O. Mujica, M. Thomas, J. Harrow, T. Cox, D. Jackson, J. Severin, P. Biggs, J. Fu, M. Nefedov, P. J. De Jong, A. F. Stewart, A. Bradley, *Nature* **2011**, *474* (7351), 337, https://doi.org/10.1038/nature10163.

[2] B. H. Kang, *BMB Rep* **2012**, *45* (1), 1, https://doi.org/10.5483/bmbrep.2012.45.1.1.

[3] N. G. Yoon, H. Lee, S.-Y. Kim, S. Hu, D. Kim, S. Yang, K. B. Hong, J. H. Lee, S. Kang, B.-G. Kim, K. Myung, C. Lee, B. H. Kang, *Journal of the American Chemical Society* **2021**, *143* (47), 19684, https://doi.org/10.1021/jacs.1c07099.

[4] OECD, *Test No. 492: Reconstructed human Cornea-like Epithelium (RhCE) test method for identifying chemicals not requiring classification and labelling for eye irritation or serious eye damage*, **2019**.
